# Inhibition of the lncRNA Coded within Transglutaminase 2 Gene Impacts Several Relevant Networks in MCF-7 Breast Cancer Cells

**DOI:** 10.3390/ncrna7030049

**Published:** 2021-08-18

**Authors:** Carlo M. Bergamini, Chiara Vischioni, Gianluca Aguiari, Carmen Grandi, Anna Terrazzan, Stefano Volinia, Nicoletta Bianchi, Cristian Taccioli

**Affiliations:** 1 Department of Neuroscience and Rehabilitation, University of Ferrara, Via Luigi Borsari, 46, 44121 Ferrara, Italy; carlo.bergamini@unife.it (C.M.B.); gianluca.aguiari@unife.it (G.A.); 2 Department of Animal Medicine, Health and Production, University of Padua, 35020 Legnaro, Italy; chiara.vischioni@phd.unipd.it (C.V.); cristian.taccioli@unipd.it (C.T.); 3 Department of Biophysical Chemistry, Institute for Molecules and Materials Radboud University, HG03.344, Heyendaalseweg 135, 6525 AJ Nijmegen, The Netherlands; carmen.grandi@ru.nl; 4 Department of Translational Medicine, University of Ferrara, Via Fossato di Mortara, 70 c.o. Viale Eliporto, 44121 Ferrara, Italy; anna.terrazzan@edu.unife.it (A.T.); stefano.volinia@unife.it (S.V.)

**Keywords:** non-coding RNA, RNA-sequencing, network, transglutaminase 2, *TGM2*

## Abstract

Long non-coding RNAs are nucleotide molecules that regulate transcription in numerous cellular processes and are related to the occurrence of many diseases, including cancer. In this regard, we recently discovered a polyadenylated long non-coding RNA (named TG2-lncRNA) encoded within the first intron of the Transglutaminase type 2 gene (*TGM2*), which is related to tumour proliferation in human cancer cell lines. To better characterize this new biological player, we investigated the effects of its suppression in MCF-7 breast cancer cells, using siRNA treatment and RNA-sequencing. In this way, we found modifications in several networks associated to biological functions relevant for tumorigenesis (apoptosis, chronic inflammation, angiogenesis, immunomodulation, cell mobility, and epithelial–mesenchymal transition) that were originally attributed only to Transglutaminase type 2 protein but that could be regulated also by TG2-lncRNA. Moreover, our experiments strongly suggest the ability of TG2-lncRNA to directly interact with important transcription factors, such as RXRα and TP53, paving the way for several regulatory loops that can potentially influence the phenotypic behaviour of MCF-7 cells. These considerations imply the need to further investigate the relative relevance of the TG2 protein itself and/or other gene products as key regulators in the organization of breast cancer program.

## 1. Introduction

### 1.1. Structure and Expression of lncRNA of Transglutaminase Type 2 Gene

The gene *TGM2* is translated into the canonical protein transglutaminase type 2 (TG2), a multifunctional enzyme regulated by different allosteric effectors and involved in various cellular activities. It catalyses intracellular reactions of Ca^2+^-dependent transamidation or of hydrolysis of GTP, as a G-protein interacting with transmembrane receptors in cell signalling, whereas outside the cell this enzyme promotes the stabilization of the ECM (Extracellular Matrix) [1,2,3,4]. These interactions are crucial in the control of cell motility and invasion, in the onset of inflammation and fibrosis, and in angiogenesis and wound repair. In addition, in nucleus it can modify structural and regulatory proteins.

The complex *TGM2* gene codes for several protein variants, along with two long non-coding RNAs (lncRNAs), one containing the last three exons of the gene and part of the 3′UTR region (AK126508), whereas the other includes a lncRNA of 1000 bp (TG2-lncRNA) located within the intron 1 (XR_001754586.1). In particular, TG2-lncRNA (https://www.ncbi.nlm.nih.gov/gene/107987281, accessed on 21 June 2021), located downstream *TGM2* first exon, is constituted by two untranslated exons produced after *TGM2* undergoes splicing. Furthermore, its sequence presents both short interspersed nuclear elements and MIR repeats. The TG2-lncRNA shows homologies with retrotransposons [5], but, given the loss of its promoter, is consequently unable to migrate to other genomic regions.

Molecular modelling indicates that this lncRNA likely forms secondary structures [5], producing duplex regions with consensus sequences recognized by transcription factors and proteins engaged in chromatin remodelling. Accordingly, RNA immunoprecipitation (RIP) identifies RNApol II, Retinoid X Receptor alpha (RXRα), MAX, and GATA3 as TG2-lncRNA associated factors [5]. Recently, TG2-lncRNA has also been linked to phosphorylated UPF-1, which is involved in nonsense-mediated decay, leading to degradation of lncRNA molecules [6].

The expression of TG2-lncRNA is under the control of the same promoter of *TGM2* and is modulated by drugs capable of increasing the levels of TG2 protein. For example, all-*trans* retinoic acid (ATRA) is used for the treatment of promyelocytic leukaemia HL-60 cells [7] and its effects on gene expression were also observed in human leukemic NB4 and neuroblastoma SK-N-SH cell lines [1]. In this context, its nuclear location and the ability to bind regulatory factors suggest a role as a possible transcriptional modulator [7].

Additional regulatory mechanisms of *TGM2* expression include epigenetic modifications through the H3K27Ac histone mark, which likely enhances the transcription of *TGM2*, whose patterns of histone acetylation and methylation are reported to be sensitive to ATRA in supporting terminal cell differentiation [8]. Methylation involves an exact RARα binding site, which is 43 base pairs upstream the ATG translation start site, affecting RAR-RXR heterodimer formation. These regulatory effects are consistent with what observed in NB4 cells, in which PML-RARα fusion protein usually represses retinoid effects, changing the differentiation program to promote cell immortalization [8].

### 1.2. TGM2 Expression in Breast Cancer

The enzyme is expressed in BrCa (breast cancer), likely with a correlation with the invasion power. Epigenetic modification at the level of the gene promoter, has also been investigated for years in tumour samples expressing *TGM2* [9]. In fact, TG2 is upregulated in intraductal invasive BrCa, also showing an increased localization in ECM, whereas normal tissues display lower expression and phenotypic differences at tumour boundaries [9]. In addition, high levels of TG2 in stroma correlate with the risk of recurrence and predict poor prognosis for patients [10]. The *TGM2* promoter shows statistically a significant association with the levels of DNA methylation and transcriptional activity, as demonstrated by Ai et al. [11]. Accordingly, the downregulation of TG2 produces an attenuated stemness in MDA-MB-231 and MCF-7, as observed recently [12].

Furthermore, the use of molecules blocking the catalytic activity of TG2 in a combined therapy with mTORC1 complex inhibitors demonstrated that this gene can be a potential target to limit drug-resistant phenotypes [13]. Inhibitors of TG2 have also been employed to prove the enzyme involvement in epithelial–mesenchymal transition (EMT), and in the invasion processes, representing effective molecules able to control oncogenic behaviours [14].

Finally, to test whether TG2-lncRNA can activate or repress specific molecular pathways, we used MCF-7 cells, which represent a suitable model to study TG2-lncRNA modulation due to the low levels of gene expression shown for this specific lncRNA. This approach has allowed a detailed observation of the correlation between TG2-lncRNA and its predicted targets and the interactions with important transcription factors involved in cancer initiation and progression. To investigate the role of TG2-lncRNA we have also performed experiments to silence its expression, using a specific short interfering RNA (siRNA) molecule verifying its biological effects using RNA-sequencing (RNA-seq) techniques, gene ontology, and network protein functional analysis.

## 2. Results

### 2.1. Downregulation of TG2-lncRNA Using a siRNA Molecule Changes Specific Pathways in MCF-7 Cancer Cells

**Figure 1:**
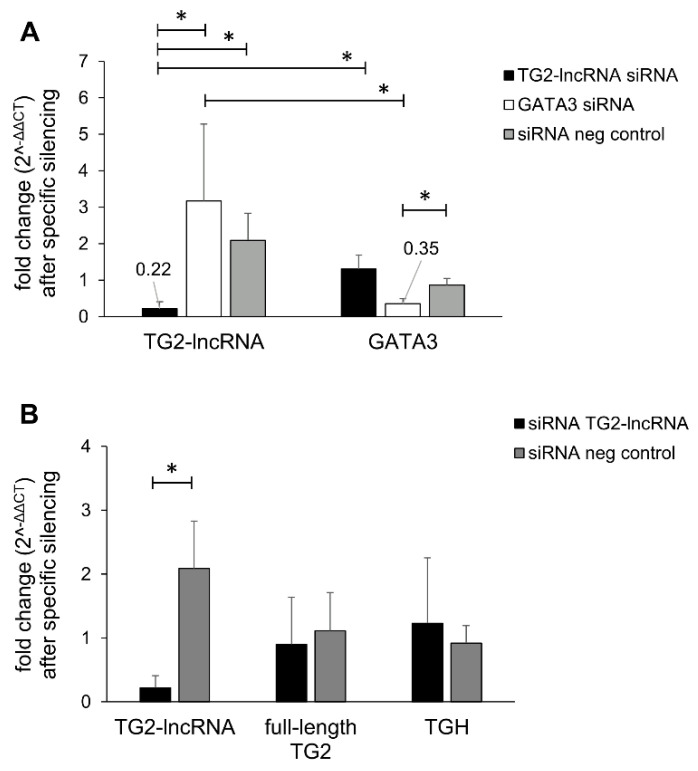
Silencing of TG2-lncRNA. The data show RT-qPCR analysis after 48 h of treatment with siRNAs employed at a concentration of 50 nM. Black, siRNA targeting TG2-lncRNA; White, siRNA directed against GATA3; Grey, control treatment with siRNA neg. In (**A**,**B**), the histograms indicate Fold Change obtained using 2^−∆∆CT^ method, referred to HPRT1 as housekeeping gene to quantify target genes with respect to the set of samples treated with siRNA neg. Error bars represent standard deviation (*n* = 3 biological replicates). * *p* < 0.05 by two-tailed unpaired *t* test.

To observe the effect and the biological functions of TG2-lncRNA silencing, we used MCF-7 cells as model (Figure 1 and Figure S1), which are known not to express *TGM2* at high levels [11,12]. Thus, we took advantage of siRNA molecules to target specifically TG2-lncRNA and GATA3 (as a positive control) transcripts in comparison with the siRNA negative control (siRNA neg), using reverse transcription and quantitative polymerase chain reaction (RT-qPCR). Figure 1A shows that the siRNA specifically designed against TG2-lncRNA selectively decreases its levels after 48 h of incubation, whereas the siRNA against GATA3 does not produce any transcriptional interference on TG2-lncRNA, although it inhibits GATA3 amplification. In addition, to verify if the exposure of the cells to siRNA against TG2-lncRNA also abolished TG2 transcripts by affecting pre-mRNA molecules, we analysed the levels of full-length TG2 and TGH which are the most expressed isoforms in MCF-7 cells. Figure 1B shows that the siRNA downregulates TG2-lncRNA without interfering with the expression of *TGM2* and its TG2 or TGH transcripts.

**Figure 2:**
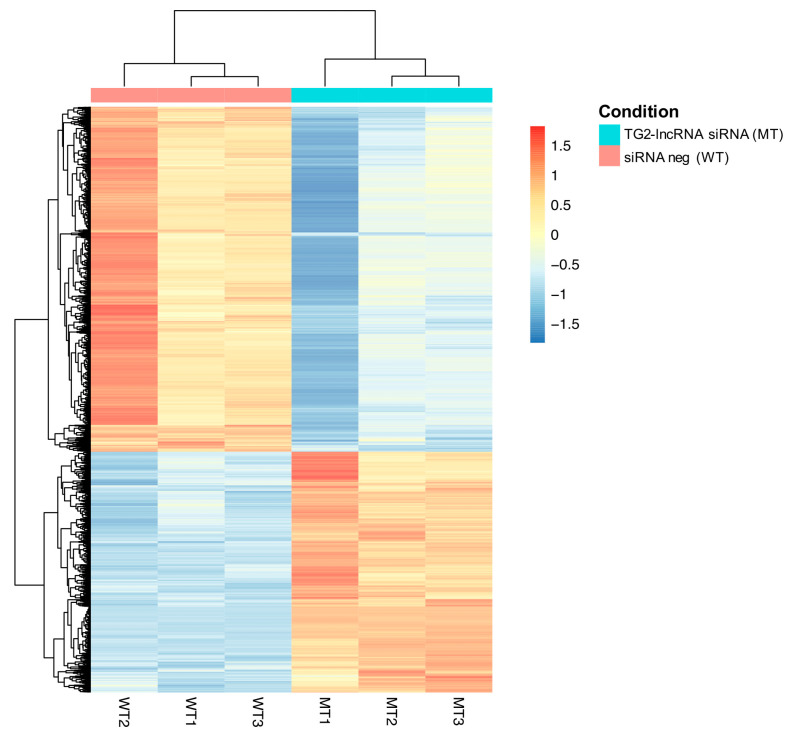
Expression heatmap of dysregulated genes in response to TG2-lncRNA silencing. “MT” group represents the treated class with siRNA against the TG2-lncRNA, whereas “WT” represents the siRNA neg control. The level of expression of the genes was obtained through DESeq2 R package, and is represented as Log_2_FC, filtered out based on *p*-adj < 0.01. The red/blue colour bar highlights the Log_2_FC rate variation across six samples for each gene. The upregulated are showed as red, whereas the downregulated genes are labelled in blue.

Furthermore, we verified the effects on gene expression profiling through RNA-seq experiments on MCF-7 cells treated with siRNA targeting TG2-lncRNA, in order to pinpoint differentially expressed genes, triggered by fluctuations of this lncRNA. Using the gene expression data of the most deregulated genes (DEGs), we created a heatmap (Figure 2), a principal component analysis (PCA) graph, and a MA-plot (Supplementary Material S1) in order to show which samples cluster together. The results show that the treated samples were grouped together and well separated from the samples used as controls, reporting, respectively, the samples clustering and the genes expression for each genotype. The number of fragments per kilobase of transcript per million mapped reads (FPKM) were filtered for FPKM > 1.

**Figure 3:**
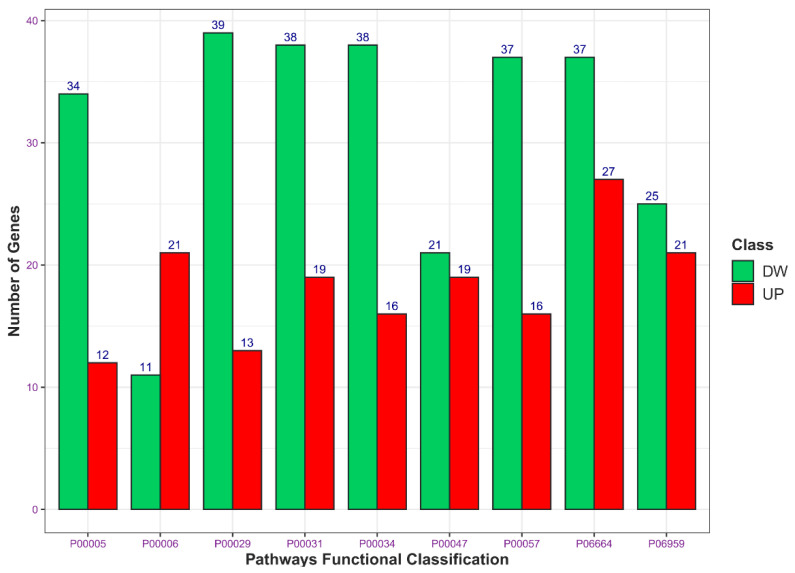
Functional classification of the most represented dysregulated pathways. The top 9 highly dysregulated pathways obtained using PANTHERdb classification system (http://www.pantherdb.org/, accessed on 21 June 2021) are indicated with the relative functional classification number, which is depicted below the bar charts, where Red and Green bars represent respectively upregulated and downregulated pathways. This analysis derives from Table S2 data (*p*-adj < 0.05). The height of each bar is proportional to the total number of dysregulated genes within the related pathway, as further labelled on the top of them.

Moreover, among a total number of 17188 analysed genes (Supplementary Material S2), 3924 genes were significantly dysregulated (*p*-adj < 0.05). Since Log_2_ Fold Change (Log_2_FC) measures the logarithmic changes in the gene expression profile due to the treatment, we considered as significantly upregulated (*n* = 1620) those genes with a positive Log_2_FC > 1.5, and significantly downregulated (*n* = 2304) those genes with a negative Log_2_FC value < 1.5. Similarly to RT-qPCR results, RNA-seq analysis did not show a significant dysregulation of *TGM2* isoform (Log_2_FC = 0.22). Moreover, we used Panther (http://pantherdb.org, accessed on 21 June 2021) to perform a gene ontology analysis, based on network functional classification. In total, 617 upregulated genes were found to be involved in 119 upregulated canonical pathways, whereas 937 downregulated genes were included in 126 downregulated ones. Among the upregulated enriched families, we observed: (1) the gonadotropin-releasing hormone receptor pathway (P06664); (2) the apoptosis signalling pathway (P00006); (3) the cholecystokinin receptor (CCKR) signalling network (P06959); (4) the platelet-derived growth factor (PDGF) signalling pathway (P00047); and (5) the inflammation mediated by chemokine and cytokine signalling molecular network (P00031). The downregulated genes, instead, were found to be involved in: (1) Huntington’s disease (P00029); (2) the integrin signalling pathway (P00034); (3) the inflammation mediated by chemokine and cytokine signalling pathway (P00031); (4) the gonadotropin-releasing hormone receptor pathway (P06664); (5) the Wnt signalling pathway (P00057); and (6) the angiogenesis molecular network (P00005). Both upregulated and downregulated genes belonging to these enriched biological families were then used to construct a network analysis (Figure 3 and Supplementary Material S3, available online at the link https://www.networkanalyst.ca, accessed on 21 June 2021)

In this study we have considered only the relevant pathways related to BrCa. Indeed, some of them are purely ascribable to other pathological contexts, such as Huntington associated genes, modulation of gonadotropin-releasing hormone receptor or CCKR signalling and Wnt signalling pathways.

**Figure 4:**
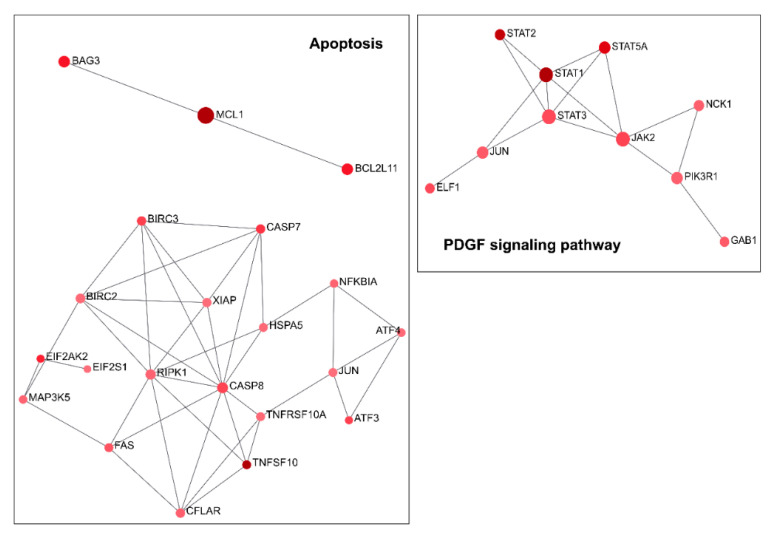
Networks of the genes upregulated by silencing of TG2-lncRNA involved in the apoptosis and PDGF signalling pathways. Each node represents one of the differentially expressed genes. In the left are depicted 2 networks belonging to apoptosis, while in the right panel the only one relevant for PDGF signalling. Red color indicates that all genes are upregulated by silencing. The node size is proportional with the degree of association with the altered gene expression value based on the Log_2_FC after siRNA treatment, while intensity of the node color scales the weight of connections. Gene correlated to each other are linked by lines.

**Figure 5:**
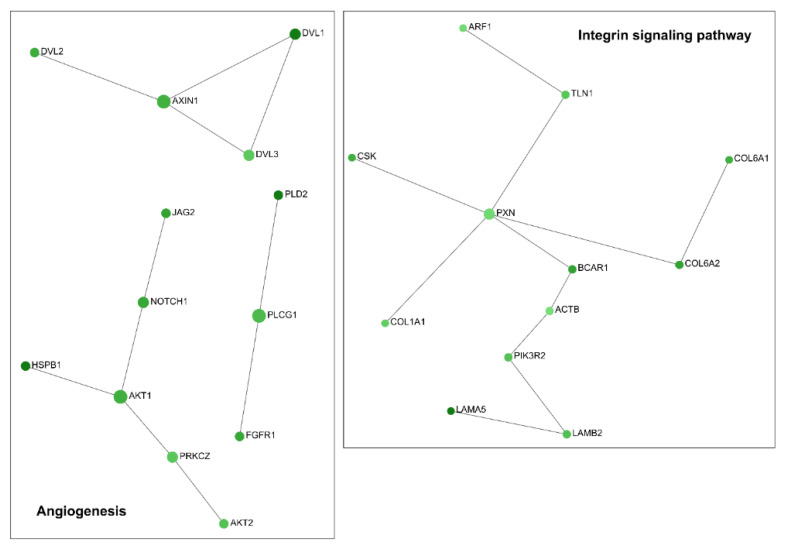
Networks of the genes downregulated by silencing of TG2-lncRNA involved in the angiogenesis and integrin signalling pathways. Each node represents one of the differentially expressed genes. In the left chart are depicted 3 networks belonging to angiogenesis, while in the right panel is reported the only one of integrin signalling. Green color indicates that all genes are downregulated by silencing. The node size is proportional with the degree of association with the modified gene expression value based on the Log_2_FC after siRNA treatment, while intensity of the node color scales the weight of connections. Gene correlated to each other are linked by lines.

**Figure 6:**
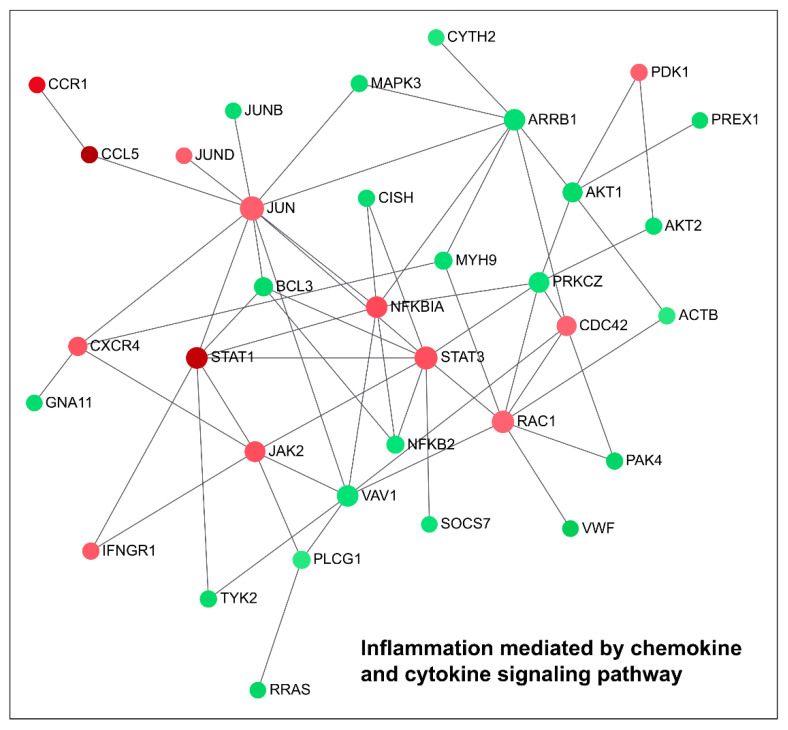
Network of the genes dysregulated by the silencing of TG2-lncRNA belonging to inflammation pathways mediated by chemokine and cytokine signalling. Each node represents one of the differentially expressed genes. In red depicted are upregulated genes, while in green, those downregulated. The node size is proportional with the degree of association with the modified gene expression value based on the Log_2_FC after siRNA treatment, while intensity of the node colour scales the weight of connections. Gene correlated to each other are linked by lines.

Moreover, this analysis highlights how the modulation of a specific siRNA against TG2-lncRNA affects processes in which *TGM2* is involved. In fact, as shown in a recent paper describing the role of *TGM2* in cancer [15], this gene is implicated in PDGF signalling, inflammation, integrin signalling, as well as angiogenesis, as we have found in our Network analysis. Indeed, our results show that apoptosis and PDGF genes are upregulated (Figure 4 and Figure 6), whereas angiogenesis and integrin genes are downregulated by silencing. Inflammation genes are instead a mixed group of upregulated (some of themSTAT1, STAT3, NFĸBIA) and downregulate genes (Figure 5, as NFĸB2). In particular, the network controlling inflammation mediated by chemokine and cytokine signalling includes genes that are well known as important transcription factors related to chronic inflammation, such as JUN (AP-1) and NF-ĸB inhibitor alpha (NFĸBIA) that are upregulated (Figure 6), whereas NFĸBI2 is downregulated as previously described. In this case, TG2-lncRNA acts both as oncogene and oncosuppressor.

The contribution of these genes (Figure 4, Figure 5 and Figure 6) to tumour progression appears obvious for the network of apoptosis (Figure 4), since the treatment with siRNA stimulates tumour necrosis factor ligand superfamily member 10 and 10A, whose interaction with receptors promotes programmed cell death through IĸB kinase/NF-ĸB signalling. Therefore, their increase, along with that of NFĸBIA, caspases 7 and 8, and tumour necrosis factor receptor superfamily member 6 (TNFRSF6), tends to promote the apoptotic processes upon TG2-lncRNA silencing. These considerations were checked by the assay of Annexin V exposition using TG2-lncRNA-silenced cells. As reported in Figure S2, we found an increase of early (after 48 h) and late (after 72 h) apoptotic cells, with comparable values of total apoptosis in both evaluations. Moreover, we observed an increase of E3 ubiquitin-protein ligase XIAP, which is well-known to organize the degradation of proteins interacting with caspases during apoptosis. Another dysregulated gene, Baculoviral IAP repeat-containing protein, shows ligase activity similar to that of XIAP. This gene also presents a relevant activity as coactivator of the eIF2, whose expression is higher after siRNA silencing, analogously to the pro-apoptotic factor Bcl-2-like protein 11 and the cyclic AMP-dependent transcription factor ATF3. The latter is a leucine-zipper protein, forming heterodimers with other members of the CREB/ATF family, like JUN, which plays an important role in the repression of interleukin and cytokine genes to contrast excessive inflammatory response. Another network we found, including overexpressed genes by silencing, is PDGF signalling. Among these we underline STAT genes (STAT1, STAT3) that mediate the effects of cellular responses to interferons, cytokines and growth factors and the downregulated (JUN, JUND in Figure 6) that act on development and tumour progression. The 3-phosphoinositide-dependent protein kinase 1 (PDK1, in Figure 6), which is known to negatively modulate TGF-β-mediated inflammation and, positively angiogenesis, is up-regulated by silencing (~25%), whereas its targets AKT1 and AKT2 (in Figure 5) appear to be downregulated (40% and 30% respectively). Notably, PDK1 is also involved in molecular adhesion and AKT1/2 usually regulate negatively endothelial cell proliferation, migration and angiogenic sprouting (Figure 5). Additional evidence that the siRNA against TG2-lncRNA disrupts functions associated with cell migration derives from the observation of a decrease of genes engaged in integrin signalling, including the Beta-actin (ACTB) and Paxillin (PXN) (as reported in Figure 5) that influence cell motility and integrin binding together, with the attachment at sites of cell adhesion to ECM.

To validate the RNA-seq and gene ontology analysis we performed RT-qPCRs on selected genes, AKT1 and Beta-actin, because of their involvement in BrCa tumourigenesis, especially in MCF-7 cells, where they act as key regulators of EMT homeostasis and STAT1, STAT2 and STAT5A of the PDGF signalling pathway. Our results showed that STAT1, STAT2 and STAT5A were increased in silenced samples compared with controls, whereas AKT1 and Beta-actin were downregulated, as reported in Supplementary Figure S3. Note that TG2-lncRNA resulted completely silenced in treated samples while TG2 does not appear to change significantly. These data confirm the trend of modulation of PDGF signalling by TG2-lncRNA silencing, whose modulation might involve cancer initiation or progression activating or inhibiting important genes, and highlight how several phases of carcinogenesis [16] might involve chronic inflammation dysregulation (Figure 6).

### 2.2. Consensus Sequences Shared between TG2-lncRNA and Promoters of TG2-lncRNA Targeted Genes

**Figure 7:**
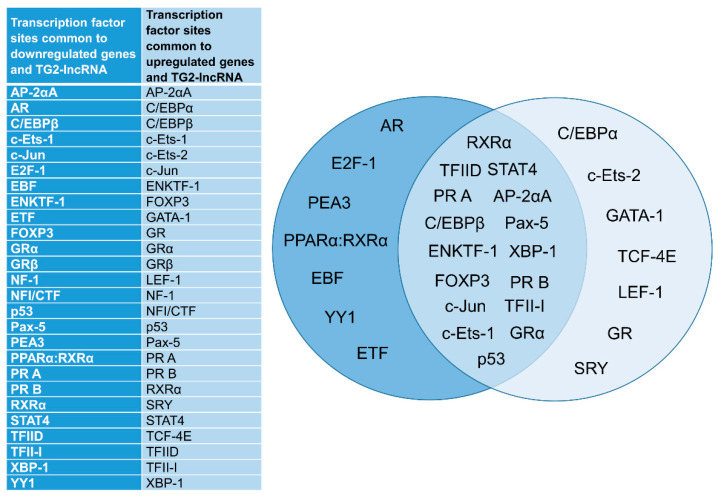
Sequences recognized by transcription factors within promoters of genes dysregulated by silencing and common to TG2-lncRNA. The left panel lists the sites within the genes downregulated and upregulated by siRNA that are also present in the sequence of TG2-lncRNA. In the right panel they are divided in three groups: dark blue, transcription factor sites common to downregulated genes also identified in TG2-lncRNA; medium blue, those common to all dysregulated genes and TG2-lncRNA; light blue, sites common to upregulated genes also identified in TG2-lncRNA.

The genes dysregulated by TG2-lncRNA silencing were analysed for the presence of consensus sites recognized by the most important transcription factors involved in cancer and present in the sequence of TG2-lncRNA. We performed this task using PROMO Alggen tool (http://alggen.lsi.upc.es/cgi-bin/promo_v3/promo/promoinit.cgi?dirDB=TF_8.3, accessed on 21 June 2021) by considering the promoters (size = 1k bp) of the most deregulated genes (first 20 up–and downregulated genes) derived by RNA-seq analysis and used them as input for PROMO. The tool returned the putative binding sites for specific transcription factors having more than 85% of similarity. The same analysis was performed for TG2-lncRNA and the results were compared in order to identify the common consensus transcription factor motives between our deregulated genes and TG2-lncRNA. Interestingly, we found 26 common regulatory elements that are summarized in Figure 7. We selected some of them to verify the possible match with TG2-lncRNA. For example, the consensus sequences of RXRα and Ying-Yang 1 (YY1) factors were present in the promoters of all the 20 downregulated genes, whereas both 20 downregulated and 20 upregulated genes shared common sequences for TP53. In particular, RXRα and TP53 were shown to share ten consensus sequences with TG2-lncRNA, while only six consensus sequences matched with YY1 genomic elements. These bioinformatic results showed the hypothetical involvement of cancer key regulators with TG2-lncRNA within a possible MCF-7 cells oncogenic environment.

### 2.3. A Biotinylated Single Strand DNA Mimic of TG2-lncRNA Binds RXRα and TP53 in Nuclear Extracts of MCF-7 Cells

Since TG2-lncRNA presents consensus sequences recognized by RXRα and is associated to this transcriptional factor in RIP complexes from NB4 cells [5], we chose it to set up in vitro binding assay. RXRα is a nuclear receptor protein that regulates the activation of numerous genes by binding with retinoic acid. Specifically, these receptors exert their action by binding to specific sequences in the promoters of target genes regulating their transcription [5]. In order to perform an in vitro binding assay, we decided to work with a more stable molecule, producing a single strand molecule of biotinylated DNA mimicking TG2-lncRNA. Two fragments of TG2-lncRNA were reconstituted using a forward biotinylated primer through the denaturation of a specific double strand molecule, recovering the products of this process using streptavidin magnetic beads, to obtain molecules capable to simulate a stable secondary structure of a single strand sequence corresponding to TG2-lncRNA. Theoretic folding of these molecules and generation of long base-paired tracts were validated in silico using an RNA secondary structure tool (http://rna.urmc.rochester.edu/RNAstructureWeb/, accessed on 21 June 2021). Among 20 possible structures, one showed long double stranded stable portions.

Thus, we tested the ability of this probe to interact with transcription factors in vitro. For this purpose, we used nuclear extracts of both BrCa cell lines and other control extracts for which the ability of TG2-lncRNA to bind nuclear factors in RIP experiments had already been described [5].

**Figure 8:**
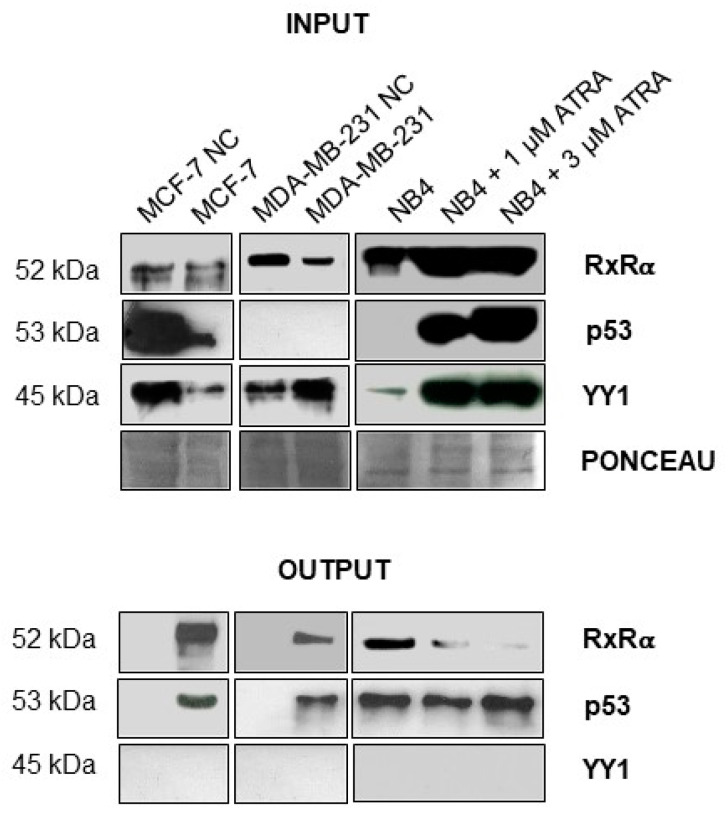
In vitro binding of RXRα, TP53 and YY1 to biotinylated single strand DNA molecule mimic of TG2-lncRNA, as detected by Western blotting of the associated protein complexes. Experimental details are reported in Material and Methods. The top picture (**INPUT**) refers to nuclear extracts (10 μL of input) from the mentioned cell lines used in each reaction of in vitro binding assay. NB4 cells represent positive control of RXRα. MCF-7 NC and MDA-MB-231 NC are control reactions in which the extracts were incubated with beads without biotinylated mimic molecule. Hybridization was carried out with specific antibody against RXRα, TP53 and YY1. In the bottom panel, we have compared (**OUTPUT**) reactions performed using the nuclear extracts after binding with the biotinylated probe. In addition, extracts from NBA cells untreated or induced with 1 and 3 μM ATRA as positive control.

Incubation of the DNA mimic of TG2-lncRNA with nuclear extracts obtained from 10^7^ MCF-7 and MDA-MB-231 cells was performed to induce in vitro assembly of protein complexes that were isolated by means of streptavidin magnetic beads. After washing they were recovered in denaturing buffer and directly loaded on polyacrylamide gel for electrophoretic separation. In parallel, we performed control experiments using extracts from leukemic NB4 cells, also induced by 1 and 3 μM ATRA, known to express both TG2-lncRNA and tested transcription factors [5]. The data reported in Figure 8 demonstrated that our probe bound protein complexes including RXRα, as evidenced by hybridization with the specific antibody. We further tested the binding of the oncosuppressor TP53, a pivotal factor involved in the regulation of the BrCa cell cycle. As shown in Figure 8 DNA mimic of TG2-lncRNA can bind both RXRα and TP53, although the former is not detectable in the extracts of MDA-MB-231 cells (in which TP53 is also mutated). Conversely, DNA mimic of TG2-lncRNA does not appear to bind YY1, despite its high binding affinity. This last observation may depend on the number of sites contained within TG2-lncRNA. In fact, as previously reported, ten consensus sequences recognized by RXRα and TP53 were present in TG2-lncRNA, whereas only six YY1 motives were contained.

## 3. Discussion

The spreading interest in the study of lncRNAs derives from their involvement in several pathologies, including cancer and neurodegenerative diseases [17,18]. In particular, lncRNAs are widely investigated in BrCa, which is the more commonly diagnosed cancer in women today [19]. For these reasons, our study aims to define the role of the recently characterized TG2-lncRNA, located within the first intron of *TGM2* gene, which is well-known to be involved in the development of various types of cancer [7]. Altered functionality of TG2 leads to the onset of diseases affecting several pathways, such as fibrosis, inflammation, and angiogenesis, and it plays a crucial role in promoting EMT and stemness phenotype, and in regulating sensitivity to chemotherapy [15,20,21].

In fact, in many tumours TG2 is expressed at higher levels compared to normal tissues, and causes tumour growth and chemoresistance, altering the responsiveness of cells to therapy [15]. Therefore, we aimed to examine if these functions should really be attributed to the TG2 protein, or, rather, to the TG2-lncRNA, which might have an additional role in controlling cell proliferation and cancer progression.

Massive scientific observations attribute variety of functional effects to the transglutaminase system [3,22]. Since TG2-lncRNA has specific localization in the nucleus and contains numerous sites for different transcription factors [5], we speculated its possible role in transcriptional regulation. Moreover, since we have recently showed that TG2-lncRNA is co-expressed with the TG2 protein [23], we decided to silence it, employing, as a model, the hormone-sensitive BrCa MCF-7 cells, in which *TGM2* expression is well characterized [23]. To this end, we used a specific siRNA against TG2-lncRNA and another siRNA molecule as a control (siRNA neg). According to Lennox and Behlke [24], strategies for silencing nuclear lncRNA should be based on antisense oligonucleotides and electroporation, using Nucleofector™ to improve nuclear delivery in suspended cells. However, we chose to silence TG2-lncRNA, a molecule 1000 bp long, using a system that had already been reported to achieve excellent results in the adherent MCF-7 cells [25]. A technological limitation to TG2-lncRNA silencing stems from the fact that there is only one suitable position to target it in a selective manner, which is the splicing site depicted in Figure S4. Consequently, it is impossible to design more than one siRNA (or one locked nucleic acid antisense molecule) to ameliorate the specificity without interfering with the pre-mRNA of *TGM2*.

Since the *TGM2* gene also leads to the accumulation of other transcriptional variants already observed in MCF-7 cells [1], we initially verified the selectivity of our siRNA against TG2-lncRNA, in order to exclude interference with other gene products. As reported above, we found 3924 genes significantly dysregulated when comparing treated cell samples versus control (three samples against three samples). The most enriched families found by our gene ontology analysis were apoptosis and PDGF signalling, which also, in this case, belong to those highly modulated by the TG2 protein [23]. Indeed, it is well-known that the inhibition of TG2 drives drug-resistant cancer cells to apoptosis through IĸBA-dependent inactivation of NF-ĸB [26], whereas the connection between TG2 and PDGF signalling can occur through E-cadherin promoting EMT [27]. NFĸBI is even a key regulator in chronic inflammation in different types of cancer [16]. Thus, we speculate that the influence of TG2-lncRNA, in these specific molecular networks, might be important in the next future when considering TG2 inhibition for therapeutic approaches.

Regarding the downregulated pathways, genes belonging to integrin signalling and angiogenesis should be mentioned. Notably, TG2 binds to proteins of ECM in order to regulate signalling mediated by the interaction with proteins such as integrins β1, β3 and β5 [3]. During carcinogenesis, the activation of specific integrin-mediated signals allows cancer cells to detach from neighbouring cells, to reorient polarization in migration, and promote survival and proliferation [28]. Silencing TG2-lncRNA led a decrease in several genes involved in the integrin pathway, which have already been reported in the literature to have a role in BrCa cell lines, including BCAR1, ITGB4, HRAS, PIK3R2, TLN1, COL1A1, ACTN4, COL5A1, MAP3K3, PXN and PIK3C2B9 [29,30,31,32,33,34,35]. Moreover, the association of TG2 with ECM is well-known to directly activate angiogenesis that is fundamental to sustain tumour metastasis [36]. This event correlates with downregulation by the silencing of TG2-lncRNA in various genes, already known to be usually overexpressed in BrCa, such as PKD1, HSPB1, DVL1, SPHK2, HRAS, JAG2, NOTCH1, AKT1, PIK3R2, DVL2, PLD2, GRB7, PDGFB, AKT2, PRKCZ, FGFR1, RHOC, PXN, PIK3C2B, and PLCG1 [35,37,38,39,40,41,42,43,44,45,46,47,48,49,50].

Another point evidenced by our study is the decrease in the levels of NFĸB2 upon treatment with siRNA in the network of chemokine/cytokine-mediated inflammation, as described also by Park et al. [50]. Although our results agree with the inhibition of inflammation through the increase in NFĸBIA, as shown previously, we also observed an increase of interleukin-15, which is important in long-term immunosurveillance against tumours [51]. Furthermore, the final balance of these effects might depend on positive- or negative-feedback molecular mechanisms involving NF-κB-dependent inflammation and TG2 activity [21]. Also, the expression of genes in this network could be highly influenced by the slight changes in gene expression levels of *TGM2*, producing relevant biological effects such as interleukin stimulation [52], cancer dissemination and metastases [53,54], and chemotherapeutic agent-resistance [26].

Moreover, the analyses using PROMO suggested that TG2-lncRNA could be involved in the recruitment of several transcription factors in order to activate or repress gene expression, driving nuclear complexes in a manner dependent on the enhancer or silencer sequences at their promoters. For example, we suppose that retinoid receptors, MAX and GATA3, as demonstrated in other types of cancer [5], could interact with the zinc finger protein YY1 that is known to modify chromatin structure and interact with insulators elements such as CTCF [55]. The experiments with a biotinylated synthetic mimic of TG2-lncRNA molecule confirmed that RXRα and TP53 can be recognized in nuclear extracts obtained from BrCa cell lines of different phenotype, such as MCF-7 or MDA-MB-231. While RXRα is known to bind TG2-lncRNA, the possibility of recognition of TP53 emerged by PROMO Alggen analysis. Thus, the expression of TG2-lncRNA during the *TGM2* activation could function as a control in the enrolment of a series of genes closely related to each other and aimed to enhance or suppress TG2 activity.

In summary, our results suggest how silencing operated by the siRNA against TG2-lncRNA leads to the activation of pathways influencing cell proliferation (PDGF) and apoptosis, while reducing angiogenesis- and integrin-controlled cell motility. These data contribute to answering the questions of the biological function of TG2-lncRNA. Our hypothesis is that it could work both like an oncogene, reducing apoptosis and increasing angiogenesis, and as a tumour suppressor, increasing integrin signalling and reducing the PDGF network. These processes may depend on the stage of cancer, reflecting a key regulatory role of TG2-lncRNA that might correlate with the activity of TG2 protein, interfering with intra and extracellular environments [56].

## 4. Material and Methods

### 4.1. Culture and Transfection of MCF-7 Cells Using siRNA Molecules

MCF-7 is a widely investigated cell line derived from invasive breast ductal carcinoma with luminal epithelial phenotype characterized by hormone sensitivity and positivity for estrogen and progesterone receptors but described as negative for human epidermal growth factor receptor-2 (HER2) [57]. The cells were cultured in Dulbecco’s modified Eagle’s medium (DMEM, Merk Life Science S.r.l., Milan, Italy), containing non-essential amino acids, 2 mM L-glutamine, 1 mM sodium pyruvate, and 1500 mg/L sodium bicarbonate, supplemented with 10% (*v*/*v*) fetal bovine serum (FBS), and antibiotic solution (50 U/mL Penicillin and 50 μg/mL Streptomycin) until an approximate density of 2–4 × 10^4^ cells/cm^2^ (70–80% confluency), at 37 °C in a humified 5% CO_2_ atmosphere. Transfection was performed with siRNA molecules by means of the Dharma FECT1RM kit (Carlo Erba Reagents, Milan, Italy) [25]. As there is no commercially available siRNA to silence TG2-lncRNA, we designed the probe to be used in the assay with the Dharmacon tool (https://horizondiscovery.com/en/ordering-and-calculation-tools/sidesign-center, accessed on 21 June 2021). The algorithm returned a series of possible molecules. Thus, we chose the compound with the best characteristics to obtain a successful silencing molecule using the correct chromosomal location across the junction between the two untranslated exons forming TG2-lncRNA genomic element, as depicted in Figure S4. This aspect is crucial to avoid silencing of the *TGM2* pre-mRNA molecule with misleading results. Hence, silencing molecules employed were: (i) siRNA (Custom XR_001754586.1 duplex) designed against the TG2-lncRNA, with 21 base lengths with the sequence 5′-GGACAAGGUUUAUGAGAUUUU-3′ and 5′-AAUCUCAUAAACCUUGUCCUU-3′ directed on the sense and antisense filament respectively; (ii) siRNA against the GATA3 mRNA (as positive control) Individual ON-TARGETplus GATA3 siRNA (cat. FE5J003781060002, Carlo Erba Reagents, Milan, Italy); and (iii) negative control siRNA (cat. FE5D0013200120, Carlo Erba Reagents, Milan, Italy). Experiments were performed in triplicate. Lyophilized siRNAs were solubilized in an RNase-free buffer (60 mM KCl, 6 mM HEPES-pH 7.5, 0.2 mM MgCl_2_) to obtain a stock concentration of 5 μM for further use in the treatment in 12-well plates (4 cm^2^/well). The day before treatment, cells were seeded at a density of 40,000 cells/cm^2^.

Two separate solutions were prepared for transfection. The first solution contained 10 μL of siRNA 5 μM and 90 μL medium without antibiotics and FBS, whereas the second solution contained 5 μL Dharma FECT1 (the transfecting agent optimal to perform with MCF-7 cells) and 95 μL medium without antibiotics and FBS. These solutions were equilibrated for 5 min at room temperature, then mixed together, to achieve a final concentration of siRNA 50 nM in the well. Then, they were mixed again and equilibrated at 25 °C for 20 min. Before treatment, culture medium was removed from each well and 800 μL of antibiotic and FBS free-medium along with 200 μL of siRNA transfection solution was added. After 8 h of incubation, cells were supplemented with 10% FBS essential for cell survival. Forty-eight hours later the supernatant was removed (to remove any residual siRNA) and cells were detached by brief treatment with 0.25% *w*/*v* trypsin, collected by centrifugation at 1200 rpm for 10 min at 4 °C and resuspended in 1 mL of Phosphate-buffered saline (137 mM NaCl, 2.7 mM KCl, 10 mM Na_2_HPO_4_, 1.8 mM KH_2_PO_4_ from Sigma-Aldrich, St. Louis, MO, USA) and centrifuged again with three additional washings.

Apoptosis was evaluated in triplicate after 48 and 72 h of culture using Muse^®^ and Annexin V & Dead Cell Kit (Luminex Corporation, Austin, TX, USA) following the procedure recommended in the user’s guide.

### 4.2. RNA Extraction, Reverse Transcription and Quantitative PCR

Cell pellets were taken up in 1 mL di Trizol^TM^ Reagent (Sigma Aldrich, St. Louis, MO, USA), lysed and RNA was purified following manufacturer’s instructions. RNA was reverse transcribed to cDNA and quantified by RT-qPCR. RT reactions were performed using TaqMan^®^ Reverse Transcription Reagents kit, following the protocol provided by Thermo Fisher Scientific with the MultiScribe™ Reverse Transcriptase, a recombinant enzyme from Murine Leukaemia Virus. The amount of RNA employed in these reactions was 1 µg, or in some cases 2 µg. The cDNA molecules obtained were further amplified by qPCR with the CFX96 Touch™ Real-Time PCR Detection System thermal cycler (Bio-rad Laboratories Srl, Segrate, Milan, Italy). Reactions were carried out by PowerUp™ SYBR^®^ Green Master Mix (Thermo Fisher Scientific, Invitrogen, Italy) in 20 µL final volume using primer pairs at the final concentration of 150 nM. Quantification was achieved through the Fold Change approach employing as reference the hypoxanthine phosphoribosyltransferase 1 (HPRT1) gene applying the formula 2^−ΔΔCT^ [58,59].

The primer pairs and PCR conditions, reported in Table S1, were designed by means of Primer-BLAST software (site http://www.ncbi.nlm.nih.gov/tools/primer-blast, accessed on 21 June 2021).

### 4.3. RNA-Sequencing and Bioinformatic Analysis

Transcriptomic analysis was performed on RNA samples of MCF-7 cells treated with siRNA against TG2-lncRNA and with siRNA negative control, as reported above. After quantification and quality checks, 1 μg of each sample was used for RNA-seq reactions carried out by BMR Genomics (www.bmr-genomics.it, accessed on 21 June 2021) (Padua, Italy), employing Next-Generation Sequencing and Illumina technology.

RNA-seq results were analysed according to the methods of Yalamanchili et al. [60], including wide spectrum reads pre-processing (quality checks and alignment) and the differential gene expression analysis, associated with visualization of results. We first used FastQC tool [61] to verify the quality of the reads, then we trimmed and removed low quality sequences by means of an awk command, built with specific parameters adapted from previous FastQC reports. Thus, we used tophat2 [62] to align the reads against the human reference genome (GRCh37, also known as hg19). After the alignment we converted the raw read counts in a table by counting the sequenced fragments [63,64], and performed the differential gene expression analysis using DESeq, which is an R package able to analyse RNA-seq data as if the read counts would follow a multinomial distribution, which can be approximated by the Poisson distribution. The package is available via Bioconductor and can be installed on R using these commands: source (“http://www.bioconductor.org/biocLite.R”) and then biocLite (“DESeq”). An easy pipeline for a two or multiclass analyses can be obtained through this command: browseVignettes (“DESeq2”) [65]. After the “two classes” analysis (treated samples *vs* controls) performed in triplicate and we only considered significant genes with adjusted *p*-value < 0.05 (Benjamini–Hochberg correction) for further analyses (Supplementary Material S2). The RNA-seq pipeline can be downloaded on GitHub of our laboratory website (https://github.com/tacclab/RNA-Seq-pipeline-TG2, accessed on 21 June 2021). In addition, the number of FPKM was calculated dividing the number of paired reads mapped to a specific gene, multiplied by kilobase and million, and divided by the total number of mapped reads × gene length in bp and filtered for FPKM > 1. Gene ontology analysis was performed with Panther (http://www.pantherdb.org/, accessed on 21 June 2021) [66]. Finally, using the output list of DEGs (most deregulated genes) by Panther, we draw the related biological network using NetworkAnalyst 3.0 web-tool [67].

### 4.4. In Vitro Assay for Binding of Nuclear Extracts by Biotinylated Single Stranded DNA Mimic of TG2-lncRNA

We have produced a molecule of biotinylated single stranded DNA corresponding to the sequence of TG2-lncRNA by PCR using two couples of primers to generate the two fragments, respectively BIOT-lncFA 5′[Biotin]-GAGTCACCGCAGCCGC-3′ and lncRA 5′-GGGGTCTTGGGGGAAAACAA-3′, lncFB 5′-CAGAGAGGAGCTTTCGGCAT-3′ and lncRB 5′-ATGAAGGTGCGCAGGGG-3′ with a thermal program 95 °C for 20 s and 60 °C for 1 min, 30 cycles. Denatured PCR products were further extended at 60 °C for 20 min. An excess of the complete biotinylated double stranded PCR product used 1 μM of concentration (10 μL) was incubated 20 min at 20 °C with 5 μL of Streptavidin Magnetic Particles (20 μg/μL, Roche) in 25 μL of Tris-EDTA buffer solution to ensure binding of double stranded PCR product. The biotinylated PCR was further denatured for 10 min at 95 °C and single stranded DNA was recovered using beads after 2 washes with warm solution, discarding the supernatant.

Biotinylated single stranded DNA mimic of TG2-lncRNA was solubilized in 100 μL of RIP buffer (25 mM Tris pH 7.5, 150 mM KCl, 5 mM EDTA, 0.5 mM DTT, 0.5% NP40, plus HALT protease inhibitor cocktail) and incubated 30 min at 4 °C in rotation with nuclear extracts [1], collected from MCF-7 and MDA-MB-231 BrCa cells. NB4 leukemic cells untreated and treated with ATRA were used as positive control, because they are known to express *TGM2*. MCF-7 culture conditions were set as reported above, while MDA-MB-231 cells were grown in DMEM (Sigma-Aldrich), 2 mM glutamine and 10% FBS in the presence of antibiotic solutions (50 U/mL penicillin and 50 μg/mL streptomycin from Sigma-Aldrich, St. Louis, MO, USA). Regarding leukemic cell cultures, treatments and preparation of nuclear extracts were performed, as described by Franzese et al. [5].

The nuclear factors bound to the biotinylated probe were recovered in association with Streptavidin magnetic beads, which were washed twice with RIP buffer. The nuclear proteins were resuspended in double concentrated denaturing buffer for Western blotting detection, with heating for 5 min at 95 °C, and the detached beads were removed by magnetic separator. Sodium dodecyl sulphate–polyacrylamide gel electrophoresis of the samples was performed on 8% gel, as reported in Franzese et al. [5], including prestained marker Sharpmass VII (Euroclone, Prestained Protein SHARPMASS™ VII Protein MW marker 6,5–270 kDa, cat. EPS026500, Pero MI, Italy). The separated proteins were transferred on Protran^TM^ 0.45 μm nitrocellulose membranes (Euroclone, Pero MI, Italy) and the hybridization was carried out using antibodies against RXRα 0.25 μg/μL (EPR7106, Abcam, Milan, Italy), TP53 (1C12, Cell signal technology, Pero MI, Italy) and YY1 (ab245365, Abcam, Abcam, Milan, Italy) employing a dilution of 1:1000 for RXRα and TP53, or dilution of 1:2000 for YY1. The bands were visualised through autoradiography and chemiluminescence system (EuroClone, Pero, MI, Italy) using HRP-conjugated secondary Ab.

## 5. Conclusions

The TG2-lncRNA is located within the *TGM2* gene, which is well-known to be associated with BrCa and is directly regulated by the same gene promoter. Given the interest of *TGM2* in oncology, we have carried out several tests in order to determine the biological function of TG2-lncRNA. The results of the analysis performed after silencing of TG2-lncRNA in MCF-7 cancer cells revealed several molecular networks in which this long non-coding RNA is potentially involved through the regulation of key genes related to apoptosis, chronic inflammation, EMT and angiogenesis. At the same time, we have shown that TG2-lncRNA binds in vitro to transcription factors recognizing the promoters of important genes, such as RXRα and TP53, which modulate the oncogenic environment of cancer cells. Finally, TG2-lncRNA appears as a double-faced biological actor because it is able to behave both like a tumour suppressor, reducing cell proliferation and increasing integrin signalling, and as an oncogene, reducing apoptosis and increasing angiogenesis. These characteristics are shared with the gene *TGM2*, which is located and transcribed in the same direction. Most of the studies carried out so far have attributed the influence of gene regulation in BrCa to the exclusive action of the TG2 protein without considering the effects of TG2-lncRNA, which should now be re-evaluated on the basis of the multiple influences it has in cancer. Its impact on our new validated pathways, of both apoptosis and PDGF signalling, underlines a subtle criticality in addressing the fate of cancer cells (death or migration), in which TG2-lncRNA expression could be an additional tool to control oncogenic processes. The simple use of TG2 enzymatic inhibitors, recently proposed as anticancer agents, may not be sufficient to have long-term benefits; consequently, it could be useful in the near future to include the TG2-lncRNA molecule as a possible target for therapeutics. On account of these promising results, we believe that the TG2-lncRNA should be studied with greater emphasis to test it as an anti-cancer drug molecule.

## Data Availability

The RNA-seq pipeline of MCF-7 cells silenced by siRNA against TG2-lcRNA versus the controls treated with siRNA negative can be downloaded on GitHub of our laboratory website https://github.com/tacclab/RNA-Seq-pipeline-TG2, 21 June 2021.

## Supplementary Materials

The following are available online at https://www.mdpi.com/article/10.3390/ncrna7030049/s1, Figure S1: Analysis of gene expression of TG2-lncRNA, TG2 and TGH transcripts in BrCa MCF-7 and MDA-MB-231 cells, Figure S2: Apoptotic rate after TG2-lncRNA silencing, Figure S3: Validation of genes modulated in PDGF signaling pathway, Figure S4: Location of TG2-lncRNA within the intron 1 of *TGM2* gene, Material S1:, Material S2: Total number of analysed genes by RNA-seq, Material S3: Up- and down-regulated pathways by silencing with TG2- lncRNA, Table S1: Primer pairs used in RT-qPCR.

## Abbreviations

ATRA	All-*trans* retinoic acid
ACTB	Beta-actin
BrCa	Breast cancer
CCKR	Cholecystokinin receptor
DEGs	Differential Gene Expression
ECM	Extracellular matrix
EMT	Epithelial–mesenchymal transition
FBS	foetal bovine serum
FPKM	number of Fragments Per Kilobase of transcript per Million mapped reads
GO	Gene ontology
HPRT1	Hypoxanthine Phosphoribosyltransferase 1
JUN	Transcription factor AP-1
lncRNAs	Long non-coding RNAs
Log_2_FC	Log_2_ Fold Change
NFĸBIA	NF-ĸB inhibitor alpha
nt	nucleotides
PCA	Principal Component Analysis
PDGF	Platelet-derived growth factor
PDK1	3-phosphoinositide-dependent protein kinase 1
PXN	Paxillin
RIP	RNA immunoprecipitation
RNA-seq	RNA sequencing
RT-qPCR	Reverse Transcription and quantitative Polymerase Chain Reaction
RXRα	Retinoid X receptor alpha
siRNA	short interfering RNA
siRNA neg	siRNA negative control
TG2	Transglutaminase type 2
TG2-lncRNA	Long non-coding RNA of Transglutaminase type 2 gene
*TGM2*	Transglutaminase type 2 gene
YY1	Ying-Yang 1
