# Supplementary material for: Inhibition of the lncRNA Coded within Transglutaminase 2 Gene Impacts Several Relevant Networks in MCF-7 Breast Cancer Cells
Source: Noncoding RNA. 2021 Aug 18;7(3):49. doi: 10.3390/ncrna7030049 (PMC8395837; doi:10.3390/ncrna7030049)
Supplement: Supplementary file 1 [file ncrna-07-00049-s001.zip › Supplementary Figures S1-S4.pdf]

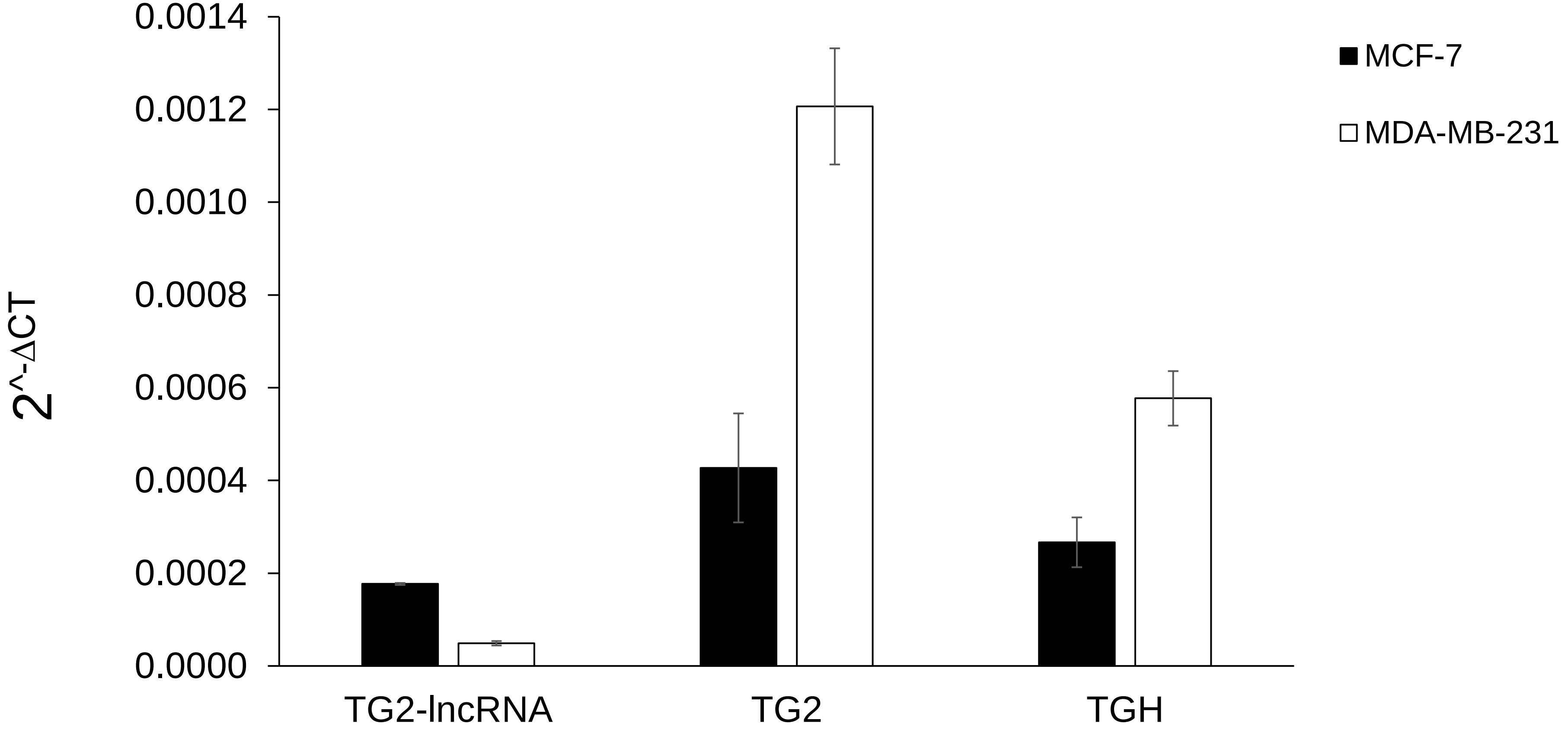

2^-ΔCT
0.0014
0.0012
0.0010
0.0008
0.0006
0.0004
0.0002
0.0000
TG2-lncRNA
TG2
TGH
MCF-7
MDA-MB-231

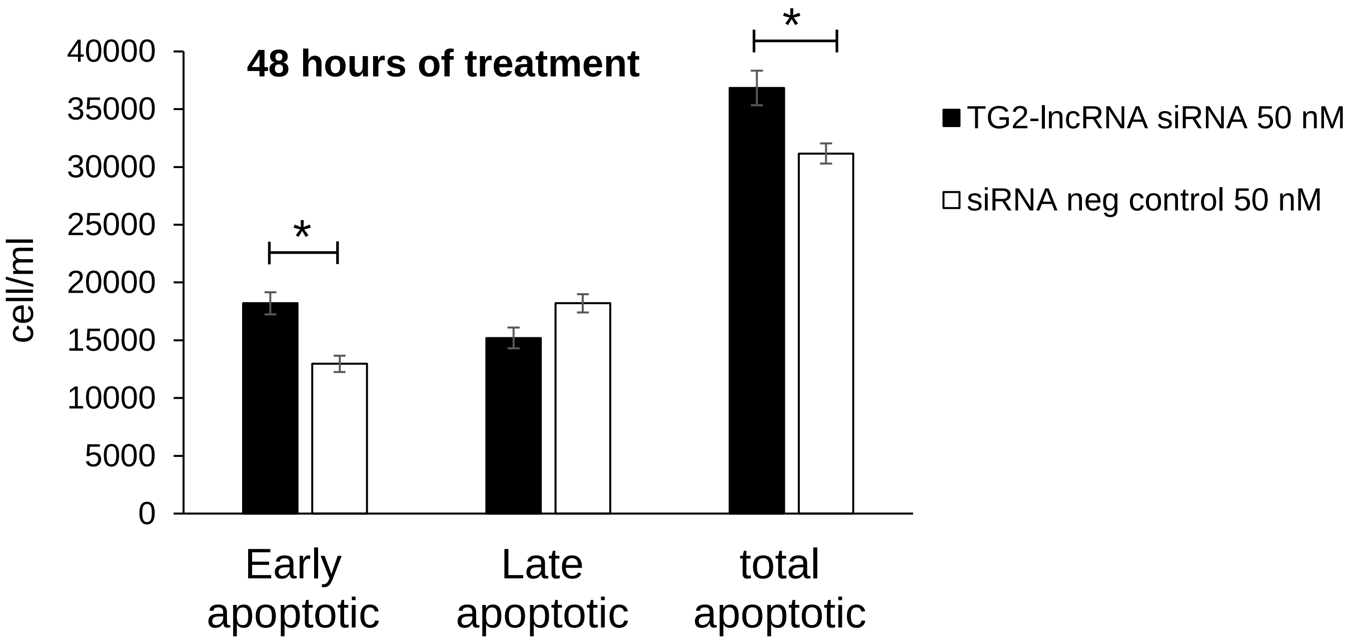
48 hours of treatment
cell/ml
40000
35000
30000
25000
20000
15000
10000
5000
0
*
*
Early apoptotic
Late apoptotic
total apoptotic
TG2-lncRNA siRNA 50 nM
siRNA neg control 50 nM

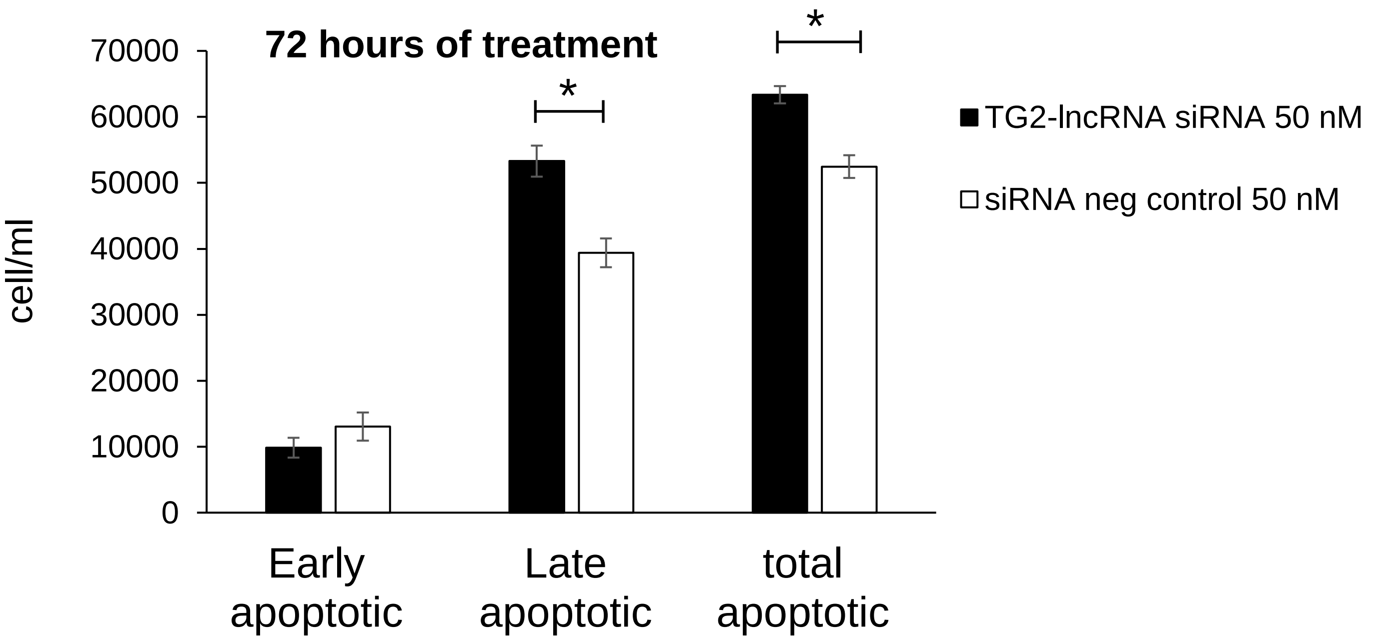
72 hours of treatment
cell/ml
70000
60000
50000
40000
30000
20000
10000
0
*
*
Early apoptotic
Late apoptotic
total apoptotic
TG2-lncRNA siRNA 50 nM
siRNA neg control 50 nM

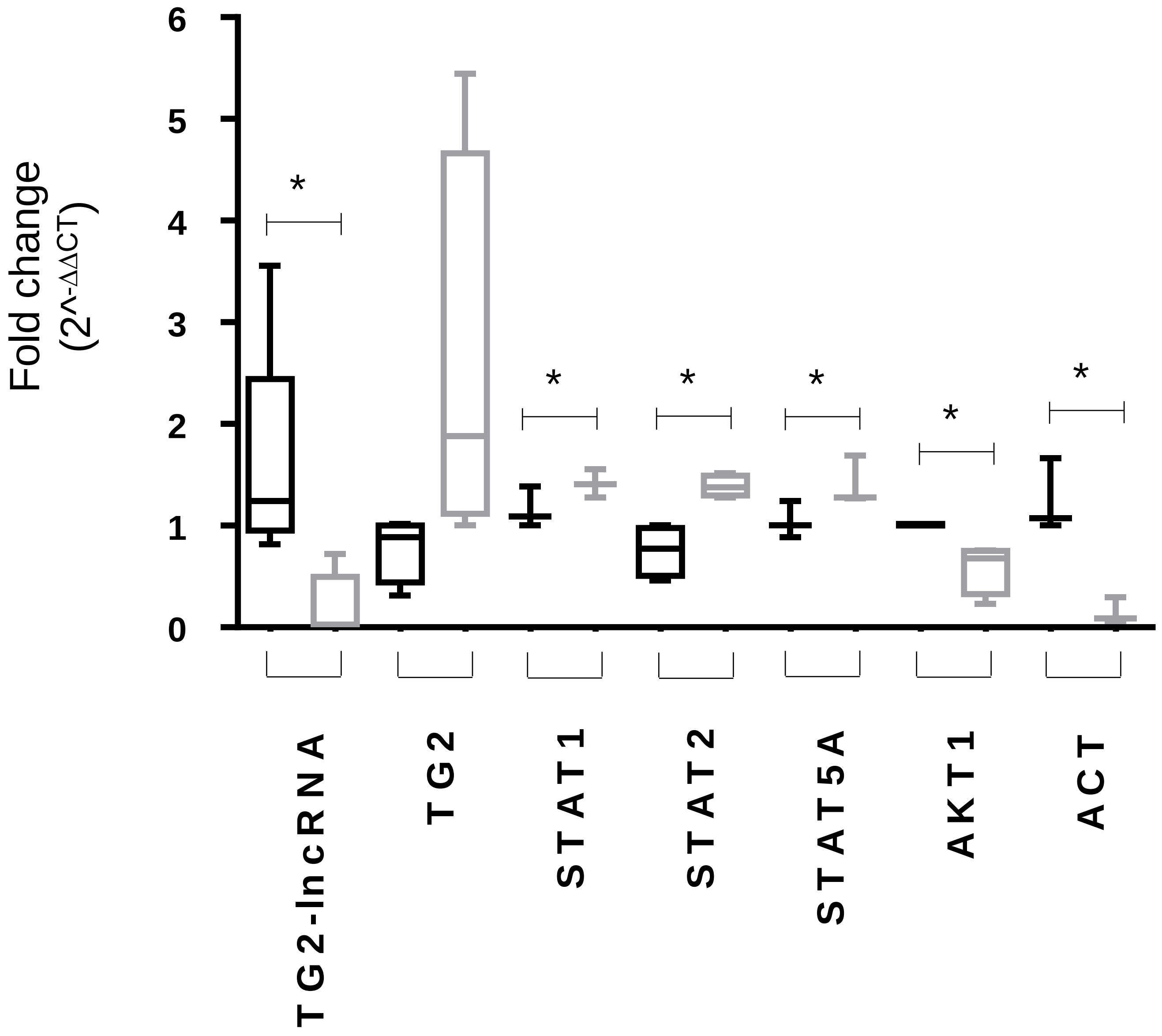

Fold change
(2^-ΔΔCT)
6
5
4
3
2
1
0
*
*
*
*
*
*
TG2-lncRNA
TG2
STAT1
STAT2
STAT5A
AKT1
ACT

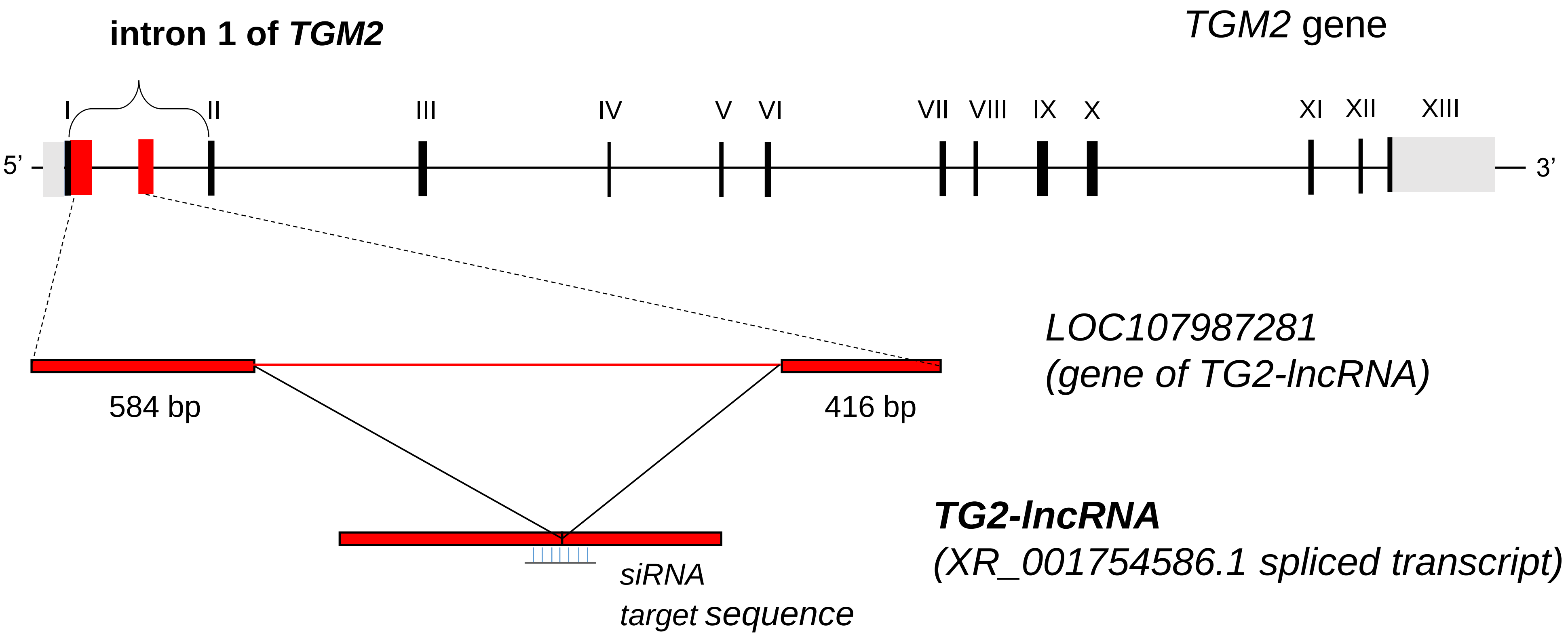
intron 1 of TGM2
TGM2 gene
I
II
III
IV
V
VI
VII
VIII
IX
X
XI
XII
XIII
5'
3'
LOC107987281
(gene of TG2-lncRNA)
584 bp
416 bp
TG2-lncRNA
(XR_001754586.1 spliced transcript)
siRNA
target sequence

Supplementary Figure 1. Analysis of gene expression of TG2-lncRNA, TG2 and TGH transcripts in MCF-7 and MDA-MB-231 BrCa cells. Two µg of total RNA of the samples were reverse transcribed. Levels of mRNA indicated were obtained using $2^{-\Delta CT}$ method to compare the levels of the amplified target genes by RT-q-PCR, and HPRT1 as reference. Error bars represent standard deviation of three independent replicates and the labels represent significantly different expression ($p<0.05$, two-tailed Unpaired t test).

Supplementary Figure 2. Apoptotic rate after TG2-lncRNA silencing. Early, late and total apoptotic cells/ml were analyzed using Annexin V assay. The measurements were performed after 48 (upper) and 72 h (lower) of treatment with 50 nM siRNA against TG2-lncRNA (Black) and negative control siRNA (White). The data represent means of 3 evaluations from 2 independent experiments ($p<0.05$ compared to negative control, two-tailed Unpaired *t* test).

Supplementary Figure 3. Validation of genes modulated in PDGF signaling pathway. Levels of the target genes (TG2-lncRNA, TG2, STAT1, STAT2, STAT5A, AKT1 and B-actin) were evaluated by RT-qPCR using as control one among the samples treated with siRNA neg. Primers and amplification conditions are reported in Supplementary Table 1. Fold Change was calculated with $2^{-\Delta\Delta CT}$ formula and HPRT1 as reference gene. In Black, are depicted samples treated with siRNA neg, while in Grey, samples treated with siRNA against TG2-lncRNA. Error bars represent standard deviation (n=3 independent experiments) and significant differences labeled (*), ($p<0.05$, Paired t test using parametric test).

Supplementary Figure 4. Location of TG2-lncRNA within the intron 1 of *TGM2* gene. The Black blocks represent the exons of *TGM2* gene, whereas the Grey ones depict the 5′ and 3′ untranslated regions. The Red blocks are the two untranslated exons that constitute TG2-lncRNA (LOC107987281). Thus, TG2-lncRNA transcript (XR_001754586.1) is obtained by the splicing and junction of these two genomic elements. The sequence recognized by the siRNA is also shown.
