# Supplementary material for: Inhibition of the lncRNA Coded within Transglutaminase 2 Gene Impacts Several Relevant Networks in MCF-7 Breast Cancer Cells
Source: Noncoding RNA. 2021 Aug 18;7(3):49. doi: 10.3390/ncrna7030049 (PMC8395837; doi:10.3390/ncrna7030049)

Supplementary Figure S1. MA plot providing the two-groups comparison. Each gene is represented as a single dot, where red dots stand for genes with p-adj value<0.01. X axis pictures represent the average expression over all samples, whereas the Y axis shows the log_2FC between treatment with siRNA against TG2-lncRNA and siRNA neg (the control).

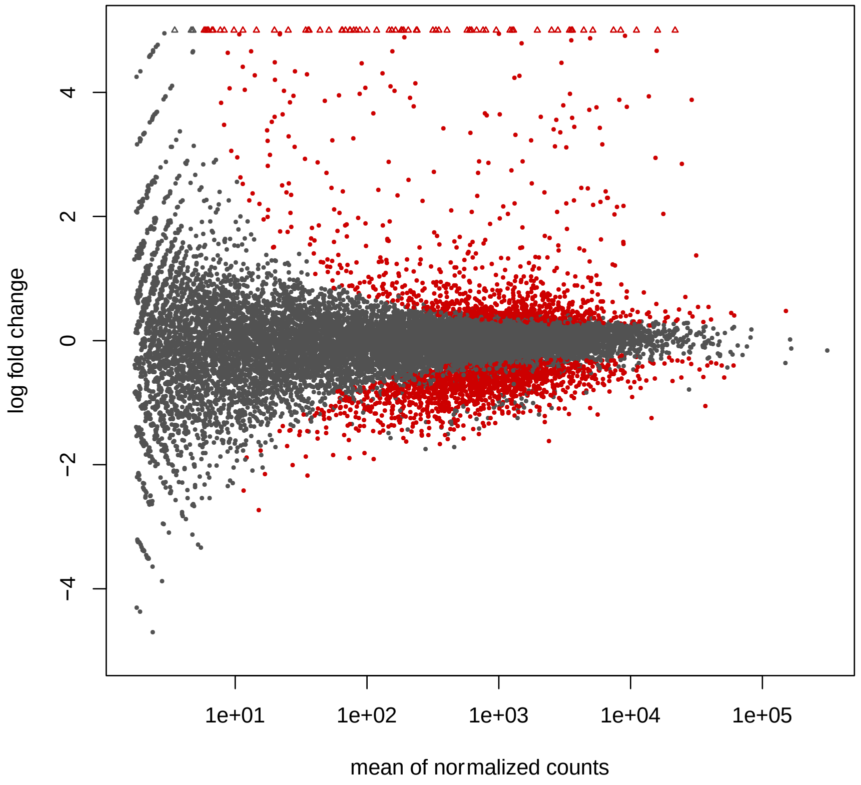

Supplementary Figure S2. Principal Component Analysis (PCA) of normalized read counts obtained from DESeq2 R package output. For each group ("MT" and "WT"), sample to sample distances comprises approximately 84% of variance, providing clustering information based on samples condition.

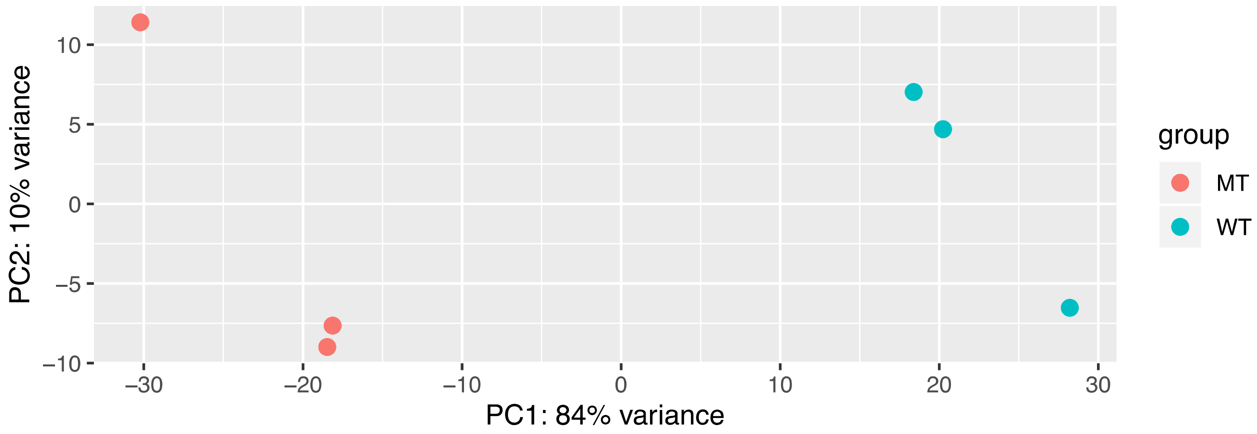

Supplement: Supplementary file 1 [file ncrna-07-00049-s001.zip › Supplementary Material S1.pdf]
