# Supplementary material for: Inhibition of the lncRNA Coded within Transglutaminase 2 Gene Impacts Several Relevant Networks in MCF-7 Breast Cancer Cells
Source: Noncoding RNA. 2021 Aug 18;7(3):49. doi: 10.3390/ncrna7030049 (PMC8395837; doi:10.3390/ncrna7030049)
Supplement: Supplementary file 1 [file ncrna-07-00049-s001.zip › Supplementary Material S3.pdf]

Supplementary Material S3. Up- and down-regulated pathways by silencing with TG2-lncRNA. The functional classification was performed using PANTHERdb web-tool (http://www.pantherdb.org/). For each highly represented pathway, the most dysregulated genes are shown, coupled with their relative Log_2FC value.

GeneID	log2FoldChange	Family/Subfamily
		Gonadotropin-releasing hormone receptor pathway
INHBA	2.425807846	Inhibin beta A chain
GNB4	1.56518265	Guanine nucleotide-binding protein subunit beta-4
KAT2B	1.542392787	Histone acetyltransferase KAT2B
ATF3	1.401037001	Cyclic AMP-dependent transcription factor ATF-3
MAP3K8	1.10551389	Mitogen-activated protein kinase kinase kinase 8
PLA2G4C	0.822500585	Cytosolic phospholipase A2 gamma
SMAD1	0.578849775	Mothers against decapentaplegic homolog 1
STAT3	0.550107469	Signal transducer and activator of transcription 3
MAP3K5	0.546003554	Mitogen-activated protein kinase kinase kinase 5
ACVR2A	0.48573124	Activin receptor type-2A
EIF2AK3	0.481983856	Eukaryotic translation initiation factor 2-alpha kinase 3
IRS2	0.471109453	Insulin receptor substrate 2
DUSP1	0.376482976	Dual specificity protein phosphatase 1
ID2	0.371542228	DNA-binding protein inhibitor ID-2
JUN	0.355541686	Transcription factor AP-1
JUND	0.351257043	Transcription factor jun-D
EGR1	0.340900989	Early growth response protein 1
BMPR2	0.33405844	Bone morphogenetic protein receptor type-2
CDC42	0.3263491	Cell division control protein 42 homolog
FOSB	0.324439134	Protein fosB
SMAD3	0.31714702	Mothers against decapentaplegic homolog 3
PIK3R1	0.285183025	Phosphatidylinositol 3-kinase regulatory subunit alpha
MAP3K13	0.269800744	Mitogen-activated protein kinase kinase kinase 13
RAC1	0.269592794	Ras-related C3 botulinum toxin substrate 1
ADIPOR2	0.264267565	Adiponectin receptor protein 2
GNAI3	0.260570907	Guanine nucleotide-binding protein G(k) subunit alpha
PEA15	0.251163921	Astrocytic phosphoprotein PEA-15
		Apoptosis signaling pathway
TNFSF10	6.173249056	Tumor necrosis factor ligand superfamily member 10
EIF2AK2	2.158342077	Interferon-induced double-stranded RNA-activated protein kinase
CASP7	1.650905351	Caspase-7
ATF3	1.401037001	Cyclic AMP-dependent transcription factor ATF-3
BIRC3	1.378687927	Baculoviral IAP repeat-containing protein 3
CASP8	1.341232621	Caspase-8
FAS	0.908303844	Tumor necrosis factor receptor superfamily member 6
CFLAR	0.733110642	CASP8 and FADD-like apoptosis regulator
MCL1	0.703922041	Induced myeloid leukemia cell differentiation protein Mcl-1
NFKBIA	0.614437952	NF-kappa-B inhibitor alpha

RIPK1	0.59073616	Receptor-interacting serine/threonine-protein kinase 1
MAP3K5	0.546003554	Mitogen-activated protein kinase kinase kinase 5
HSPA5	0.460457838	Endoplasmic reticulum chaperone BiP
BIRC2	0.441274559	Baculoviral IAP repeat-containing protein 2
TNFRSF10A	0.426137216	Tumor necrosis factor receptor superfamily member 10A
ATF4	0.390241923	Cyclic AMP-dependent transcription factor ATF-4
JUN	0.355541686	Transcription factor AP-1
XIAP	0.321299731	E3 ubiquitin-protein ligase XIAP
BAG3	0.316405178	BAG family molecular chaperone regulator 3
BCL2L11	0.312519757	Bcl-2-like protein 11
EIF2S1	0.280639445	Eukaryotic translation initiation factor 2 subunit 1
CCKR signaling map		
BIRC3	1.378687927	Baculoviral IAP repeat-containing protein 3
PRKD2	1.347771687	Serine/threonine-protein kinase D1
HBEGF	0.994881555	Proheparin-binding EGF-like growth factor
KLF4	0.958279183	Krueppel-like factor 4
MCL1	0.703922041	Induced myeloid leukemia cell differentiation protein Mcl-1
NFKBIA	0.614437952	NF-kappa-B inhibitor alpha
JAK2	0.5852736	Tyrosine-protein kinase JAK2
STAT3	0.550107469	Signal transducer and activator of transcription 3
BIRC2	0.441274559	Baculoviral IAP repeat-containing protein 2
RHEB	0.357486174	GTP-binding protein Rheb
JUN	0.355541686	Transcription factor AP-1
ELK4	0.351442404	ETS domain-containing protein Elk-4
PDK1	0.345893494	3-phosphoinositide-dependent protein kinase 1
EGR1	0.340900989	Early growth response protein 1
PKD2	0.340058515	Serine/threonine-protein kinase D2
CDC42	0.3263491	Cell division control protein 42 homolog
TEP1	0.295990766	Phosphatidylinositol 3.4.5-trisphosphate 3-phosphatase and dual-specificity protein phosphatase PTEN
EIF4E	0.295727168	Eukaryotic translation initiation factor 4E
PIK3R1	0.285183025	Phosphatidylinositol 3-kinase regulatory subunit alpha
RAC1	0.269592794	Ras-related C3 botulinum toxin substrate 1;RAC1;ortholog
YWHAB	0.232205336	14-3-3 protein beta/alpha
CDH1	0.203481454	Cadherin-1
PDGF signaling pathway		
STAT1	2.844671677	Signal transducer and activator of transcription 1-alpha/beta
STAT2	2.40513065	Signal transducer and activator of transcription 2
STAT5A	1.813281567	Signal transducer and activator of transcription 5A
PDGFRL	0.730025882	Platelet-derived growth factor receptor-like protein
EHF	0.59690827	ETS homologous factor
JAK2	0.5852736	Tyrosine-protein kinase JAK2
STAT3	0.550107469	Signal transducer and activator of transcription 3
RPS6KC1	0.533161081	Ribosomal protein S6 kinase delta-1

ARHGAP27	0.531272325	Rho GTPase-activating protein 27
ELF1	0.528234983	ETS-related transcription factor Elf-1
GAB1	0.366045108	GRB2-associated-binding protein 1
JUN	0.355541686	Transcription factor AP-1
ELK4	0.351442404	ETS domain-containing protein Elk-4
PDK1	0.345893494	3-phosphoinositide-dependent protein kinase 1
ARHGAP12	0.331602857	Rho GTPase-activating protein 12
NCK1	0.323048126	Cytoplasmic protein NCK1
SRGAP1	0.306900148	SLIT-ROBO Rho GTPase-activating protein 1
PIK3R1	0.285183025	Phosphatidylinositol 3-kinase regulatory subunit alpha
PIK3R3	0.262079328	Phosphatidylinositol 3-kinase regulatory subunit gamma
Inflammation mediated by chemokine and cytokine signaling pathway		
CCL22	4.463931916	C-C motif chemokine 22
CCL5	3.350672845	C-C motif chemokine 5
IL15	2.931601231	Interleukin-15
STAT1	2.844671677	Signal transducer and activator of transcription 1-alpha/beta
FPR3	1.920739943	N-formyl peptide receptor 3
CCR1	1.82943547	C-C chemokine receptor type 1
NFKBIA	0.614437952	NF-kappa-B inhibitor alpha
JAK2	0.5852736	Tyrosine-protein kinase JAK2
STAT3	0.550107469	Signal transducer and activator of transcription 3
CXCR4	0.518688609	C-X-C chemokine receptor type 4
GNG5	0.446618845	Guanine nucleotide-binding protein G(I)/G(S)/G(O) subunit gamma-5
IFNGR1	0.412321764	Interferon gamma receptor 1
JUN	0.355541686	Transcription factor AP-1
JUND	0.351257043	Transcription factor jun-D
PDK1	0.345893494	3-phosphoinositide-dependent protein kinase 1
CDC42	0.3263491	Cell division control protein 42 homolog
TEP1	0.295990766	Phosphatidylinositol 3.4.5-trisphosphate 3-phosphatase and dual-specificity protein phosphatase PTEN
RAC1	0.269592794	Ras-related C3 botulinum toxin substrate 1
GNAI3	0.260570907	Guanine nucleotide-binding protein G(k) subunit alpha
Integrin signalling pathway		
LAMC2	1.0641345	Laminin subunit gamma-2
RND1	0.702723654	Rho-related GTP-binding protein Rho6
MAP3K5	0.546003554	Mitogen-activated protein kinase kinase kinase 5
RND3	0.533645576	Rho-related GTP-binding protein RhoE
LAMB1	0.404323201	Laminin subunit beta-1
FN1	0.347057259	Fibronectin
PTPN12	0.334770982	Tyrosine-protein phosphatase non-receptor type 12
SLK	0.327032119	Tyrosine-protein kinase Fyn
CDC42	0.3263491	Cell division control protein 42 homolog
ELMO2	0.287811232	Engulfment and cell motility protein 2
PIK3R1	0.285183025	Phosphatidylinositol 3-kinase regulatory subunit alpha

ARF6	0.279626974	ADP-ribosylation factor 6
RAC1	0.269592794	Ras-related C3 botulinum toxin substrate 1
ARL1	0.26744603	ADP-ribosylation factor-like protein 1
PIK3R3	0.262079328	Phosphatidylinositol 3-kinase regulatory subunit gamma
CRKL	0.253468554	Crk-like protein
GeneID	log2FoldChange	Family/Subfamily

Huntington disease

GRIN3B	-1.527141118	Glutamate receptor ionotropic. NMDA 3B
GRIN1	-1.445339444	Glutamate receptor ionotropic. NMDA 1
GRIN2C	-1.120595251	Glutamate receptor ionotropic. NMDA 2C
CAPN10	-1.074198348	Calpain-10
TP63	-0.993790699	Tumor protein 63
NCOR2	-0.898026076	Nuclear receptor corepressor 2
TUBB3	-0.894214098	Tubulin beta-3 chain
RAC3	-0.880877763	Ras-related C3 botulinum toxin substrate 3
GRIN2D	-0.821548402	Glutamate receptor ionotropic. NMDA 2D
CAPN12	-0.80848351	Calpain-12
GRIK3	-0.783870575	Glutamate receptor ionotropic. kainate 3
AP2A1	-0.686463563	AP-2 complex subunit alpha-1
AKT1	-0.664943358	RAC-alpha serine/threonine-protein kinase
ARPC1B	-0.653738979	Actin-related protein 2/3 complex subunit 1B
MAP3K10	-0.643977467	Mitogen-activated protein kinase kinase kinase 10
GAPDHS	-0.62229233	Glyceraldehyde-3-phosphate dehydrogenase. testis-specific
CAPN5	-0.621990729	Calpain-5
PACSIN1	-0.60602791	Protein kinase C and casein kinase substrate in neurons protein 1
CAPN1	-0.597666462	Calpain-1 catalytic subunit
MAP2K7	-0.587577916	Dual specificity mitogen-activated protein kinase kinase 7
SIN3B	-0.575191406	Paired amphipathic helix protein Sin3b
TP73	-0.542506655	Tumor protein p73
DNAH1	-0.505631272	Dynein heavy chain 1. axonemal
BAX	-0.488672159	Apoptosis regulator BAX
DNAL4	-0.485788777	Dynein light chain 4. axonemal
AKT2	-0.469498922	RAC-beta serine/threonine-protein kinase
VAT1	-0.461990287	Synaptic vesicle membrane protein VAT-1 homolog
CYC1	-0.455620854	Cytochrome c1. heme protein. mitochondrial
TUBB4B	-0.413026507	Tubulin beta-4B chain
AP2A2	-0.405588568	AP-2 complex subunit alpha-2
CLTB	-0.364207241	Clathrin light chain B
ARF5	-0.359499384	ADP-ribosylation factor 5
DCTN1	-0.341504673	Dynactin subunit 1
ARPC1A	-0.320977045	Actin-related protein 2/3 complex subunit 1A
ARF1	-0.319753767	ADP-ribosylation factor 1
CYFIP1	-0.293103703	Cytoplasmic FMR1-interacting protein 1
TUBB	-0.273216404	Tubulin beta chain
HIP1	-0.261874709	Huntingtin-interacting protein 1

ACTB	-0.23044627	Actin. cytoplasmic 1

Integrin signalling pathway

LAMA5	-1.617190413	Laminin subunit alpha-5
BCAR1	-1.064359848	Breast cancer anti-estrogen resistance protein 1
COL6A2	-1.058182594	Collagen alpha-2(VI) chain
COL18A1	-1.049529373	Collagen alpha-1(XVIII) chain
COL9A3	-0.948836499	Collagen alpha-3(IX) chain
ITGB4	-0.936037674	Integrin beta-4
CSK	-0.887200331	Tyrosine-protein kinase CSK
COL6A1	-0.877090331	Collagen alpha-1(VI) chain
COL12A1	-0.838607177	Collagen alpha-1(XII) chain
SH3GL1	-0.836416353	Endophilin-A2;SH3GL1
HRAS	-0.807512006	GTPase Hras
ARFGAP1	-0.807081849	ADP-ribosylation factor GTPase-activating protein 1
RRAS	-0.781740215	Ras-related protein R-Ras
RND2	-0.746526739	Rho-related GTP-binding protein RhoN
COL11A2	-0.737636099	Collagen alpha-2(XI) chain
COL27A1	-0.713444202	Collagen alpha-1(XXVII) chain
LAMB2	-0.6775181	Laminin subunit gamma-1
LAMB2	-0.6775181	Laminin subunit beta-2
MAP2K2	-0.659588191	Dual specificity mitogen-activated protein kinase kinase 2
ARPC1B	-0.653738979	Actin-related protein 2/3 complex subunit 1B
PIK3R2	-0.616559673	Phosphatidylinositol 3-kinase regulatory subunit beta
ITGA2B	-0.572574536	Integrin alpha-Iib
TLN1	-0.557883692	Talin-1
MAPK3	-0.536386507	Mitogen-activated protein kinase 3
RHOB	-0.513967594	Rho-related GTP-binding protein RhoB
MICALL1	-0.47127199	MICAL-like protein 1
COL1A1	-0.467055191	Collagen alpha-1(I) chain
ACTN4	-0.458228924	Alpha-actinin-4
COL5A1	-0.444272098	Collagen alpha-1(V) chain
MAP3K3	-0.409075186	Mitogen-activated protein kinase kinase kinase 3
FLNB	-0.399801824	Filamin-B;FLNB
RHOC	-0.344791739	Rho-related GTP-binding protein RhoC
MAPK13	-0.321707445	Mitogen-activated protein kinase 13
ARPC1A	-0.320977045	Actin-related protein 2/3 complex subunit 1A
PXN	-0.320939119	Paxillin
ARF1	-0.319753767	ADP-ribosylation factor 1
PIK3C2B	-0.282797065	Phosphatidylinositol 4-phosphate 3-kinase C2 domain-containing subunit beta
ACTB	-0.23044627	Actin cytoplasmic 1

Inflammation mediated by chemokine and cytokine signaling pathway

VWF	-1.058886323	von Willebrand factor
COL6A2	-1.058182594	Collagen alpha-2(VI) chain

GNAO1	-0.950015155	Guanine nucleotide-binding protein G(o) subunit alpha
RAC3	-0.880877763	Ras-related C3 botulinum toxin substrate 3
COL6A1	-0.877090331	Collagen alpha-1(VI) chain
COL12A1	-0.838607177	Collagen alpha-1(XII) chain
RRAS	-0.781740215	Ras-related protein R-Ras
PAK4	-0.74480369	Serine/threonine-protein kinase PAK 4
GRK6	-0.741761596	G protein-coupled receptor kinase 6
PLCB3	-0.684497523	1-phosphatidylinositol 4.5-bisphosphate phosphodiesterase beta-3
BCL3	-0.666240556	B-cell lymphoma 3 protein
AKT1	-0.664943358	RAC-alpha serine/threonine-protein kinase
ADRBK1	-0.661630082	Beta-adrenergic receptor kinase 1
ARPC1B	-0.653738979	Actin-related protein 2/3 complex subunit 1B
PREX1	-0.651782791	Phosphatidylinositol 3.4.5-trisphosphate-dependent Rac exchanger 1 protein
GNB3	-0.644440001	Guanine nucleotide-binding protein G(I)/G(S)/G(T) subunit beta-3
TYK2	-0.631624035	Non-receptor tyrosine-protein kinase TYK2
CISH	-0.588017295	Cytokine-inducible SH2-containing protein
JUNB	-0.544523709	Transcription factor jun-B
GNA11	-0.544467554	Guanine nucleotide-binding protein subunit alpha-11
MYH9	-0.538916508	Myosin-9
MAPK3	-0.536386507	Mitogen-activated protein kinase 3
ARRB1	-0.533189068	Beta-arrestin-1;ARRB1
ITPR3	-0.521678468	Inositol 1.4.5-trisphosphate receptor type 3
AKT2	-0.469498922	RAC-beta serine/threonine-protein kinase
ALOX15	-0.461284628	Arachidonate 15-lipoxygenase
PRKCZ	-0.426168221	Protein kinase C zeta type
VAV1	-0.419709425	Proto-oncogene vav
INPPL1	-0.409332916	Phosphatidylinositol 3.4.5-trisphosphate 5-phosphatase 2
SOCS7	-0.382640297	Suppressor of cytokine signaling 7
SOCS7	-0.382640297	Suppressor of cytokine signaling 4
RHOC	-0.344791739	Rho-related GTP-binding protein RhoC
NFKB2	-0.344585361	Nuclear factor NF-kappa-B p100 subunit
MYLK	-0.337938238	Myosin light chain kinase smooth muscle
ARPC1A	-0.320977045	Actin-related protein 2/3 complex subunit 1A
CYTH2	-0.301795304	Cytohesin-2
PLCG1	-0.258462189	1-phosphatidylinositol 4.5-bisphosphate phosphodiesterase gamma-1
ACTB	-0.23044627	Actin. cytoplasmic 1
Gonadotropin-releasing hormone receptor pathway		
AMH	-1.022806169	Muellerian-inhibiting factor
GNAO1	-0.950015155	Guanine nucleotide-binding protein G(o) subunit alpha
DGKZ	-0.856118407	Diacylglycerol kinase zeta
MAPK12	-0.854621835	Mitogen-activated protein kinase 12
GNB2	-0.81907454	Guanine nucleotide-binding protein G(I)/G(S)/G(T) subunit beta-2
HRAS	-0.807512006	GTPase Hras
ESRRA	-0.791927125	Steroid hormone receptor ERR1

MAP4K2	-0.782468132	Mitogen-activated protein kinase kinase kinase kinase 2
CRTC1	-0.759738314	CREB-regulated transcription coactivator 1
MAP3K11	-0.753563275	Mitogen-activated protein kinase kinase kinase 11
PLA2G6	-0.728646195	85/88 kDa calcium-independent phospholipase A2
TCF3	-0.726388628	Transcription factor 7-like 1
SDF4	-0.718213976	45 kDa calcium-binding protein
GATA4	-0.685087013	Transcription factor GATA-4
AKT1	-0.664943358	RAC-alpha serine/threonine-protein kinase
MAP2K2	-0.659588191	Dual specificity mitogen-activated protein kinase kinase 2
PRKAR1B	-0.658646115	cAMP-dependent protein kinase type I-beta regulatory subunit
GNB3	-0.644440001	Guanine nucleotide-binding protein G(I)/G(S)/G(T) subunit beta-3
MAP3K10	-0.643977467	Mitogen-activated protein kinase kinase kinase 10
MAPK11	-0.600193	Mitogen-activated protein kinase 11
MAP2K7	-0.587577916	Dual specificity mitogen-activated protein kinase kinase 7
PER1	-0.570285483	Period circadian protein homolog 1
JUNB	-0.544523709	Transcription factor jun-B
GNA11	-0.544467554	Guanine nucleotide-binding protein subunit alpha-11
ADCY1	-0.537621199	Adenylate cyclase type 1
INHBB	-0.536771596	Inhibin beta B chain
MAPK3	-0.536386507	Mitogen-activated protein kinase 3
ITPR3	-0.521678468	Inositol 1.4.5-trisphosphate receptor type 3
ALOX15	-0.461284628	Arachidonate 15-lipoxygenase
PRKCZ	-0.426168221	Protein kinase C zeta type
DNM1	-0.421597215	Dynamin-1
PGR	-0.416331499	Progesterone receptor
MAP3K3	-0.409075186	Mitogen-activated protein kinase kinase kinase 3
LDB1	-0.400519621	LIM domain-binding protein 1
GATA2	-0.386205382	Endothelial transcription factor GATA-2
MAPK13	-0.321707445	Mitogen-activated protein kinase 13
PXN	-0.320939119	Paxillin
Wnt signaling pathway		
APC2	-1.168306861	Adenomatous polyposis coli protein 2
FZD2	-1.08090775	Frizzled-2
DVL1	-1.036327016	Segment polarity protein dishevelled homolog DVL-1
DVL1	-1.036327016	Putative segment polarity protein dishevelled homolog DVL1P1
LRP5	-0.986651347	Low-density lipoprotein receptor-related protein 5
KREMEN2	-0.957393419	Kremen protein 2
CSNK1G2	-0.944342478	Casein kinase I isoform gamma-2
GNG13	-0.939964974	Guanine nucleotide-binding protein G(I)/G(S)/G(O) subunit gamma-13
TLE2	-0.824544366	Transducin-like enhancer protein 2
GNB2	-0.81907454	Guanine nucleotide-binding protein G(I)/G(S)/G(T) subunit beta-2
TCF3	-0.726388628	Transcription factor 7-like 1
PCDHA3	-0.695359572	Protocadherin alpha-3
PCDHA5	-0.684954931	Protocadherin alpha-5
PLCB3	-0.684497523	1-phosphatidylinositol 4.5-bisphosphate phosphodiesterase beta-3

PCDH1	-0.668008224	Protocadherin-1
PPARD	-0.644789773	Peroxisome proliferator-activated receptor delta
GNB3	-0.644440001	Guanine nucleotide-binding protein G(I)/G(S)/G(T) subunit beta-3
B4GALT7	-0.630943238	Beta-1.4-galactosyltransferase 7
BCL9	-0.625074731	B-cell CLL/lymphoma 9 protein
KREMEN1	-0.601417862	Kremen protein 1
DVL2	-0.590275263	Segment polarity protein dishevelled homolog DVL-2
SMARCA4	-0.557239697	Transcription activator BRG1
CELSR1	-0.550420168	Cadherin EGF LAG seven-pass G-type receptor 1
GNA11	-0.544467554	Guanine nucleotide-binding protein subunit alpha-11
AXIN1	-0.542558775	Axin-1
ARRB1	-0.533189068	Beta-arrestin-1
PCDH19	-0.526369617	Protocadherin-19
LRP5L	-0.524758537	Low-density lipoprotein receptor-related protein 5-like protein
PCDH7	-0.524236384	Protocadherin-7
ITPR3	-0.521678468	Inositol 1.4.5-trisphosphate receptor type 3
AES	-0.500060358	TLE family member 5
PRKCZ	-0.426168221	Protein kinase C zeta type
DVL3	-0.341295119	Segment polarity protein dishevelled homolog DVL-3
CTBP1	-0.296273507	C-terminal-binding protein 1
TLE3	-0.265267952	Transducin-like enhancer protein 3
ARID1A	-0.251612731	AT-rich interactive domain-containing protein 1A
ACTB	-0.23044627	Actin. cytoplasmic 1
	Angiogenesis	
PKD1	-1.247281967	Serine/threonine-protein kinase D1
HSPB1	-1.194113008	Heat shock protein beta-1
APC2	-1.168306861	Adenomatous polyposis coli protein 2
FZD2	-1.08090775	Frizzled-2
DVL1	-1.036327016	Segment polarity protein dishevelled homolog DVL-1
DVL1	-1.036327016	Putative segment polarity protein dishevelled homolog DVL1P1
EFNB2	-0.926870711	Ephrin-B2
SPHK2	-0.842281094	Sphingosine kinase 2
HRAS	-0.807512006	GTPase Hras
JAG2	-0.770567304	Protein jagged-2
NOTCH1	-0.74020498	Neurogenic locus notch homolog protein 1
AKT1	-0.664943358	RAC-alpha serine/threonine-protein kinase
MAP2K2	-0.659588191	Dual specificity mitogen-activated protein kinase kinase 2
EPHB3	-0.651298154	Ephrin type-B receptor 3
PIK3R2	-0.616559673	Phosphatidylinositol 3-kinase regulatory subunit beta
EFNB1	-0.602478098	Ephrin-B1
DVL2	-0.590275263	Segment polarity protein dishevelled homolog DVL-2
PLD2	-0.585104698	Phospholipase D2
AXIN1	-0.542558775	Axin-1
GRB7	-0.536691043	Growth factor receptor-bound protein 7
PDGFB	-0.536644982	Platelet-derived growth factor subunit B

MAPK3	-0.536386507	Mitogen-activated protein kinase 3
MAPK3	-0.536386507	MAP kinase-activated protein kinase 3
RHOB	-0.513967594	Rho-related GTP-binding protein RhoB
SCAP	-0.49474368	SH2 domain-containing protein 2A
AKT2	-0.469498922	RAC-beta serine/threonine-protein kinase
PRKCZ	-0.426168221	Protein kinase C zeta type
FGFR1	-0.359581042	Fibroblast growth factor receptor 1
RHOC	-0.344791739	Rho-related GTP-binding protein RhoC
DVL3	-0.341295119	Segment polarity protein dishevelled homolog DVL-3
BIRC5	-0.329225606	Baculoviral IAP repeat-containing protein 5
PXN	-0.320939119	Paxillin
PIK3C2B	-0.282797065	Phosphatidylinositol 4-phosphate 3-kinase C2 domain-containing subunit beta
PLCG1	-0.258462189	1-phosphatidylinositol 4.5-bisphosphate phosphodiesterase gamma-1
