# Supplementary material for: Inhibition of the lncRNA Coded within Transglutaminase 2 Gene Impacts Several Relevant Networks in MCF-7 Breast Cancer Cells
Source: Noncoding RNA. 2021 Aug 18;7(3):49. doi: 10.3390/ncrna7030049 (PMC8395837; doi:10.3390/ncrna7030049)
Supplement: Supplementary file 1 [file ncrna-07-00049-s001.zip › Supplementary Table S1.pdf]

Supplementary Table S1. Primer pairs used in RT-qPCR.

Primer	Sequence	PCR size (bp)	PCR conditions
HPRT1F<br>HPRT1R	5’-TGACACTGGCAAAACAATGCA-3’<br>5’-GGTCCTTTTCACCAGCAAGCT-3’	94	50°C 2 min<br>95°C 10 min<br>45 cycles:<br>95°C 20 sec<br>60°C 1 min
lncRNA F<br>lncRNA R	5’-TCACGTTCTGCAACCCTAGAC-3’<br>5’- GGGGTCTTGGGGGAAAACAAA-3’	117	50°C 2 min<br>95°C 10 min<br>45 cycles:<br>95°C 20 sec<br>60°C 1 min
TGM2Fex11-12<br>TGM2Rex11-12	5’-CCTTACGGAGTCCAACCTCA-3’<br>5’-CCAGCTTGCGTTTCTGCTTG-3’	152	50°C 2 min<br>95°C 10 min<br>45 cycles:<br>95°C 15 sec<br>60°C 1 min
Fshort3ex<br>Rshort3ex	5′-TCATGAGCACCCTCCTTTCT-3′<br>5′-GGTCCATTCTCACCTTAACTTCCT -3’	377	50°C 2 min<br>95°C 10 min<br>45 cycles:<br>95°C 15 sec<br>62°C 1 min
F2TGH<br>F2TGH	5’-TACAAATACCCAGAGGGGTCC-3’<br>5’-ATGTTGTCAGTTGGCGGTCA-3’	338	50°C 2 min<br>95°C 10 min<br>45 cycles:<br>95°C 30 sec<br>62°C 1 min
FGATA3Ex<br>RGATA3Ex	5'-CAGCACAGAAGGCAGGGAG-3'<br>5'-TCTGACAGTTCGCACAGGAC-3'	197	50°C 2 min<br>95°C 10 min<br>45 cycles:<br>95°C 20 sec<br>60°C 1 min
F-STAT1<br>R-STAT1	5’-TGTTATGGGACCGCACCTTC-3’<br>5’-CTCATTCACATCTCTCAACTTCACA-3’	152	50°C 2 min<br>95°C 10 min<br>45 cycles:<br>95°C 20 sec<br>60°C 1 min
F-STAT2<br>R-STAT2	5’-GCCAAAGGGAAGACACCCTC-3’<br>5’-AGCTGCCTCAGGTGAAACAA-3’	284	50°C 2 min<br>95°C 10 min<br>45 cycles:<br>95°C 20 sec

			60°C 1 min
F-STAT5A<br>R-STAT5A	5’-GAAGATCAAGCTGGGGCACT-3’<br>5’-CGGACCAGCCTCTGTTCATT-3’	114	50°C 2 min<br>95°C 10 min<br><u>45 cycles:</u><br>95°C 20 sec<br>60°C 1 min
F-AKT1<br>R-AKT1	5'-GCGTGACCATGAACGAGTTT-3'<br>5'-CTTGGCCACGATGACTTCCT-3'	211	50°C 2 min<br>95°C 10 min<br><u>45 cycles:</u><br>95°C 20 sec<br>60°C 1 min
FB-ACT<br>RB-ACT	5'-CATTGCCGACAGGATGCA-3'<br>5'-GCTGATCCACATCTGCTGGA-3'	151	50°C 2 min<br>95°C 10 min<br><u>45 cycles:</u><br>95°C 20 sec<br>60°C 1 min
RPL13aF<br>RPL13aF	5’-GAAGATGGCGGAGGGGCAG-3’<br>5’-ATGCCTTCACAGCGTACGACC-3’	126	50°C 2 min<br>95°C 10 min<br><u>40 cycles:</u><br>95°C 30 sec<br>62°C 1 min
